# Heterologous expression and characterization of functional mushroom tyrosinase (*Ab*PPO4)

**DOI:** 10.1038/s41598-017-01813-1

**Published:** 2017-05-12

**Authors:** Matthias Pretzler, Aleksandar Bijelic, Annette Rompel

**Affiliations:** Universität Wien, Fakultät für Chemie, Institut für Biophysikalische Chemie, Althanstraße 14, 1090 Wien, Austria

## Abstract

Tyrosinases are an ubiquitous group of copper containing metalloenzymes that hydroxylate and oxidize phenolic molecules. In an application context the term ‘tyrosinase’ usually refers to ‘mushroom tyrosinase’ consisting of a mixture of isoenzymes and containing a number of enzymatic side-activities. We describe a protocol for the efficient heterologous production of tyrosinase 4 from *Agaricus bisporus* in *Escherichia coli*. Applying this procedure a pure preparation of a single isoform of latent tyrosinase can be achieved at a yield of 140 mg per liter of autoinducing culture medium. This recombinant protein possesses the same fold as the enzyme purified from the natural source as evidenced by single crystal X-ray diffraction. The latent enzyme can be activated by limited proteolysis with proteinase K which cleaves the polypeptide chain after K382, only one The latent enzyme can amino acid before the main *in-vivo* activation site. Latent tyrosinase can be used as obtained and enzymatic activity may be induced in the reaction mixture by the addition of an ionic detergent (e.g. 2 mM SDS). The proteolytically activated mushroom tyrosinase shows >50% of its maximal activity in the range of pH 5 to 10 and accepts a wide range of substrates including mono- and diphenols, flavonols and chalcones.

## Introduction

**Figure 1:**
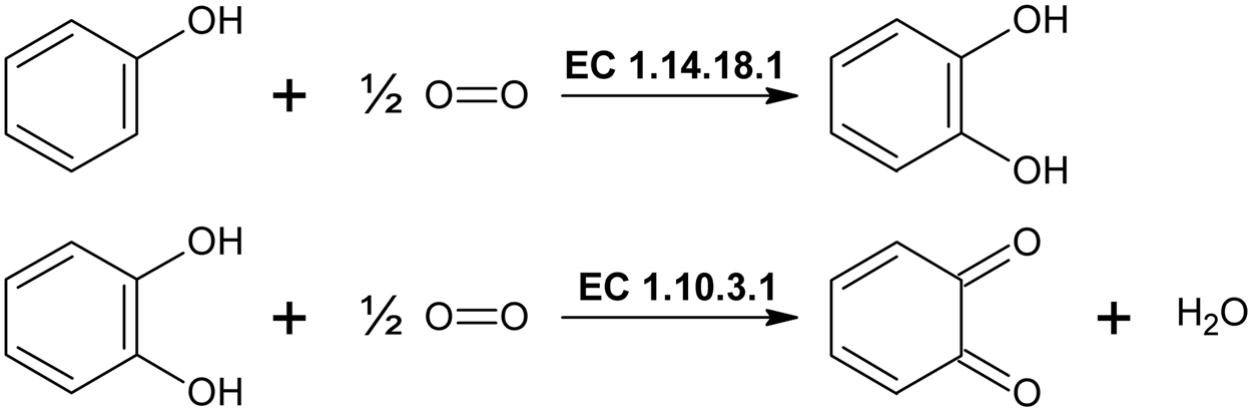
Tyrosinase catalyzes the *o*-hydroxylation of monophenols (top) and the oxidation of *o*-diphenols (bottom).

Tyrosinases form an ubiquitous family of metalloenzymes and are found in all domains of life^1^. They are of central importance for the pigmentation in vertebrates as the reactions catalyzed by tyrosinase provide the starting material for melanin biosynthesis^2^. Tyrosinases catalyze the *ortho*-hydroxylation of monophenols to *o*-diphenols (monophenolase or cresolase activity, EC 1.14.18.1) as well as the subsequent two-electron oxidation to the respective *o*-quinones (diphenolase or catechol oxidase activity, EC 1.10.3.1), which is coupled with the reduction of molecular oxygen to water^3, 4^. The active site of tyrosinase is composed of two copper ions which are coordinated by three histidine side chains each^5^ forming a type III copper center^6^. Activation of molecular oxygen is affected by binding of dioxygen to the type III copper center in a characteristic ‘side-on’ bridging mode (µ-η^2^:η^2^)^7–9^. Hydroxylation and oxidation of one monophenol to the corresponding *o*-quinone requires one molecule of O_2_, while two *o*-diphenols can be oxidized to *o*-quinones per molecule of O_2_ consumed (see Fig. 1).

The products of the reactions catalyzed by tyrosinase – *o*-quinones – are highly reactive and consequently commonly unstable in the biological environment they are generated in^10^. Therefore, they do participate in a number of non-enzymatic, spontaneous reactions. The best studied among those reactions are the formation of high-molecular-weight adducts, especially melanin^10–14^, Michael-type nucleophilic 1,4-additions^15, 16^ and the direct coupling of two quinones (‘phenol coupling’)^15, 17, 18^. As tyrosinases can oxidize both small phenolic molecules and phenolic moieties of larger molecules (e.g. proteins) they have found a plethora of biotechnological applications in e.g. organic synthesis, determination of phenolic analytes, bioremediation as well as in medicine, food processing and engineering of (bio)materials^19, 20^.

Most of these application utilize tyrosinase isolated from fruiting bodies of the common white mushroom *Agaricus bisporus* (‘mushroom tyrosinase’)^20^, supposedly mainly due to its ready commercial availability^21^. However, it should be noted that the purification protocols used to prepare the available commercial preparations do usually not yield homogenous tyrosinase and these are therefore likely to contain one or more unspecified ‘extras’ like laccase, β-glucosidase, β-xylosidase, cellulase, chitinase and xylanase activities^22, 23^. The purity of these preparations is further compromised by the fact that *Agaricus bisporus* possesses genes coding for six different tyrosinases (*Ab*PPO1 - *Ab*PPO6)^24, 25^. Of those six genes, at least two (*Ab*PPO3 and *Ab*PPO4)^25–27^ are expressed in significant amounts in the fruiting bodies which serve as the source material for the commercial preparations of ‘mushroom tyrosinase’. Considering these limitations of the widely used tyrosinase preparations in terms of both purity and batch-to-batch variability, an alternative enzyme source that manages to provide a single isoform of tyrosinase in a pure form and at a constant quality would promote both basic (protein-)biochemical research and biotechnological applications of tyrosinase. Herein, we describe such a protocol, yielding pure *Ab*PPO4 in its latent form as well as providing access to the mature, active form of the enzyme.

## Results

### Sequence of *Ab*PPO4

The initial PCR on the *A*. *bisporus* cDNA with primers enframing the ORF for *Ab*PPO4 (*Ab*PPO4_fwd and *Ab*PPO4_rev) yielded two distinct bands at 1.8 kbp (expected size of the gene) and 1.4 kbp (Figure S3). By cloning and sequencing both molecules were identified as the expected gene for *Ab*PPO4. While the larger one (1836 bp) contained the complete gene encoding amino acids M1 to F611, the smaller band (1401 bp) was missing 435 bp corresponding to 145 amino acids. The loss of these bases occurred in such a manner that the reading frame was conserved and amino acids A436 to A580 were deleted from the translation product. This deletion starts in the middle of exon number 6 and spans the last intron as well as more than two-thirds of the last exon in the gene for *Ab*PPO4. On the protein level the missing amino acids are all located in the C-terminal domain and contain the CXXC-motif as well as approximately half of the putative trans-membrane helix^27^. Expression of this construct was attempted but for all the conditions tested the heterologous protein was found exclusively in the insoluble fraction and no tyrosinase activity could be detected in the cell lysate.

With respect to the published sequence for the *Ab*PPO4-gene^28^ the cloned full-length gene contains 23 mutations among which four are non-silent (see Table 1). Of those two are located in the main domain and two at the end of the C-terminal domain. The two variations in the main domain are already known to be compatible with enzymatic activity as they were also encountered in the enzyme purified from the natural source^27^. Those and the two remaining changed amino acids as well as all the changes to the amino acid sequence in the second sequence (with the exception of the deletion of 145 amino acids) are predicted to be non-detrimental to the enzyme’s function by SIFT^29^. Expression of the full-length construct (up to F611) confirms this prediction as the heterologous enzyme was found to be fully active.

**Table 1:** Sequences of the cloned genes.

Sequence	Mutations relative to GQ354802.1^# 28^
*Ab*PPO4 full length	C21T, T168C, T306C, **T362C** (**V121A**), T483C, A504C, **G536A** (**S179N**), A540C, T717C, T735C, G1089A, C1104T, C1131G, G1218A, C1359T, T1449C, C1458T, A1521G, C1650T, T1686C, T1704C, **G1717A** (**V573I**), **G1783A** (**A595T**)
*Ab*PPO4 Δ(A436-A580)	C21T, **G97A** (**V33I**), **G133T** (**A45S**), T168C, **G301A** (**V101I**), G324C, **T362C** (**V121A**), T483C, A504C, **G536A** (**S179N**), A540C, **G563A** (**R188K**), C618T, **C620G** (**A207G**), T171C, T735C, G1089A, C1131G, **T1172A & C1173A** (**L391Q**), **G1783A** (**A595T**)

^#^The sequences are given as mutations relative to GQ354802 (mRNA for the reference sequence for *Ab*PPO4, Uniprot: C7FF05) with non-silent mutations shown in bold and followed by the respective altered amino acid in parentheses. Sequence numbers start with 1 at the A of the start codon and M of the peptide chain for nucleobases and amino acids, respectively.

### Expression and purification of *Ab*PPO4

The latent form of the tyrosinase (up to T565)^27^ was expressed as a fusion protein with an N-terminal tag, namely glutathione-S-transferase from *Schistosoma japonicum* ^30^. Initial expression attempts employing induction by addition of IPTG yielded a big amount of heterologous protein which was however almost exclusively in an insoluble form. Cultivation at lower temperatures and the use of autoinduction medium^31^ did produce a very small fraction of fusion protein in soluble form so that enzymatic activity could be detected in the cell lysate after 3 days of reaction. The addition of 500 mM NaCl to the cultivation medium did decrease the specific growth rate of the production strain by 30% causing a marked increase in total cultivation time but did also increase the fraction of soluble protein by several orders of magnitude. This expression protocol as described in Methods yields around 200 mg of fusion protein per liter of medium after capture and purification by affinity chromatography employing the affinity of GST to glutathione immobilized on cross-linked agarose. Removal of the fusion partner by the specific protease HRV 3 C was usually quite efficient with yields between 80% and 100% corresponding to approximately 110 to 140 mg of latent tyrosinase per liter of expression culture. The preparations were still active after 1 year of storage at 4 °C in the used 10 mM HEPES pH 7.5 buffer. For long-term storage freezing in liquid nitrogen^32^ is recommended as the preparations tend to darken after a few months of storage. This darkening, however, does not cause the loss of enzymatic activity.

While the full-length construct did behave almost identical as the latent version of *Ab*PPO4 in heterologous expression, the construct encoding only the main domain of the tyrosinase (up to S383)^27^ did not exhibit any tyrosinase activity.

### Activation of latent *Ab*PPO4

**Figure 2:**
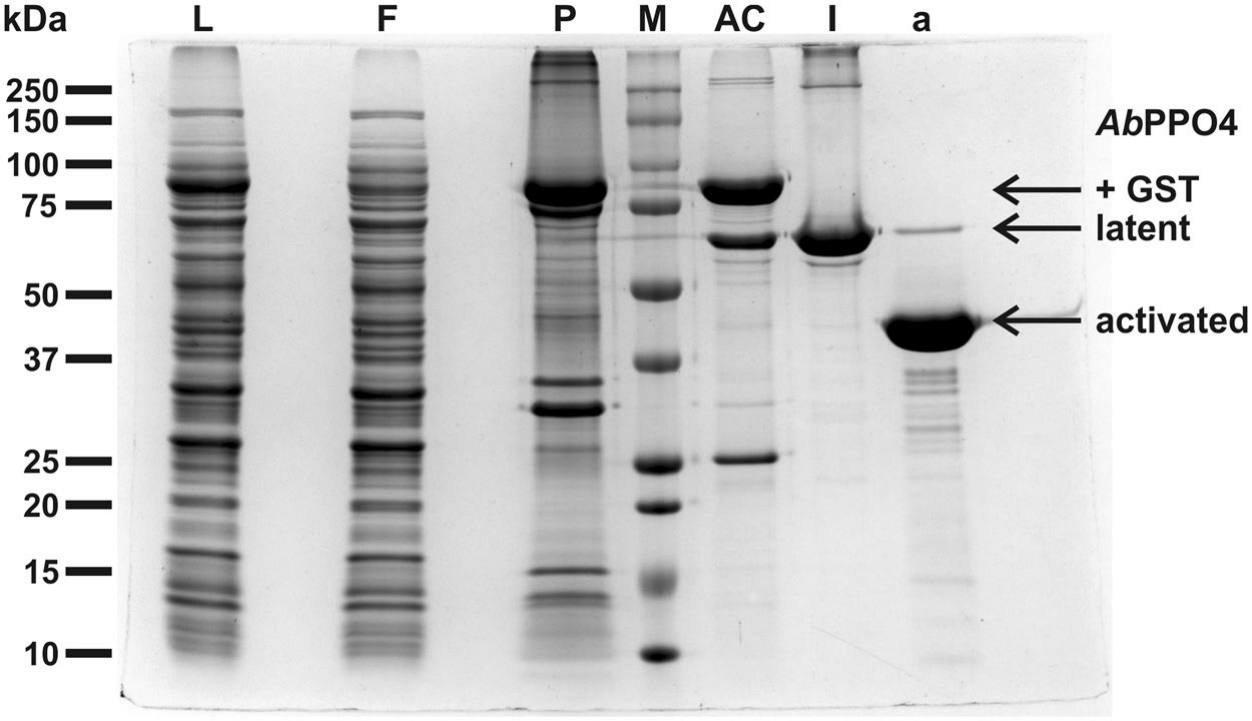
SDS-PAGE (10–15**%** acrylamide) of the four purification stages. **L**: cell lysate, **F**: flow-through of the first affinity chromatography step, **P**: insoluble cell material, **M**: molecular weight marker; Weights of the used standard protein are indicated left of the gel., **AC**: eluate of the first affinity chromatography step, **l**: latent *Ab*PPO4 (eluate of the second affinity chromatography step), **a**: activated *Ab*PPO4 (eluate of size exclusion chromatography after activation with proteinase K).

Proteolytic activation of the latent tyrosinase was tested with trypsin and proteinase K and both proteases induced tyrosinase activity in the latent enzyme. The activation by proteinase K was more efficient and did yield an essentially pure preparation of active tyrosinase while even after prolonged incubation with trypsin a second species of approximately 52 kDa remained in the reaction mixture (Figure S4). The digestion with an unspecific protease resulted in a low yield of the activation of 25–50% corresponding to 19–34 mg per liter of culture media. The activated protein appeared as a single peak in the size exclusion chromatogram and had a purity of >95% as estimated from SDS-PAGE (Fig. 2).

**Figure 3:**
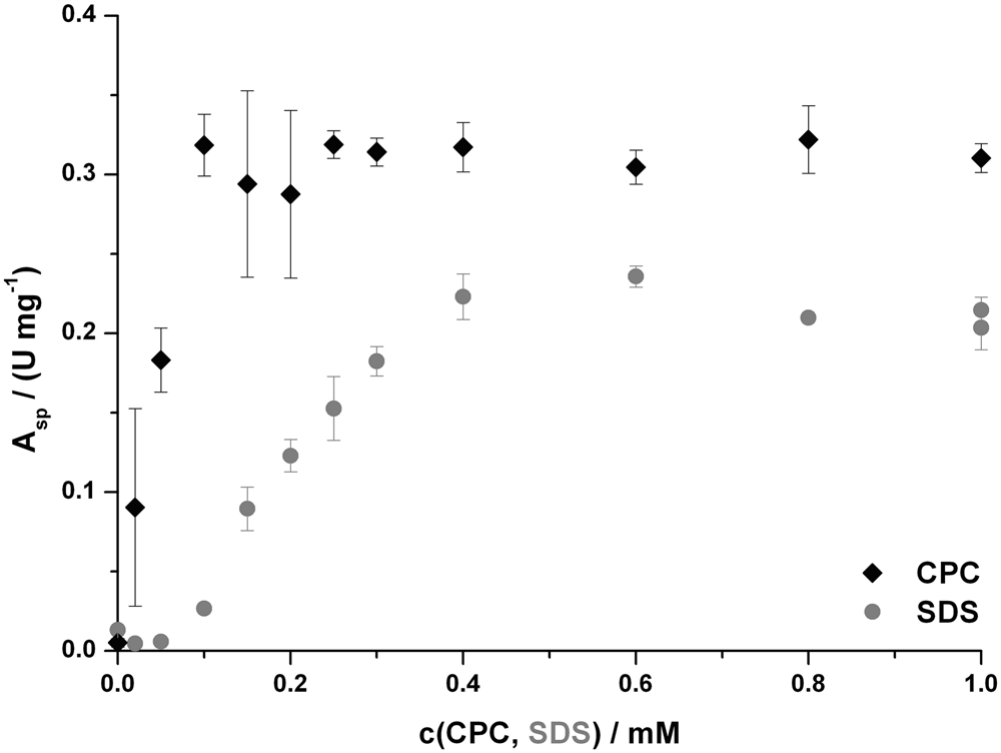
Activation of latent *Ab*PPO4 by ionic detergents. One data point represents the average of three activity measurements on 1 mM *L*-tyrosine with the indicated concentration of detergent in the respective assay mixture. The error bars indicate ± one standard deviation.

Enzymatic activity could be induced in the latent tyrosinase by treatment with ionic detergents. The cationic detergent cetylpyridinium chloride (CPC) was more effective in activating latent *Ab*PPO4 than the anionic sodium dodecyl sulfate (SDS). Maximal activation was achieved by applying 0.1 mM of CPC and reached a level which was 25% higher than that at the optimal SDS-concentration of 0.6 mM (see Fig. 3). The activity level for activation by CPC remained fairly constant at concentrations higher than 0.1 mM but above 2 mM the latent tyrosinase was prone to precipitation. For SDS the enzymatic activity decreased slightly above 0.6 mM but remained constant at circa 90% of the maximal level for up to 5 mM SDS. Above this concentration the enzymatic activity did slowly decrease but no protein precipitation was observed even at SDS levels above 100 mM.

### Analysis of the purified tyrosinase by intact protein mass spectrometry

**Figure 4:**
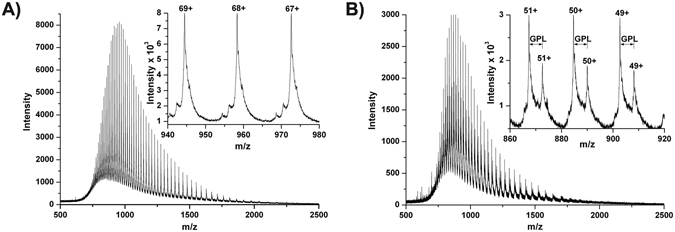
ESI-MS of latent and activated *Ab*PPO4; (**A**) latent *Ab*PPO4, (**B**) activated *Ab*PPO4.

**Figure 5:**
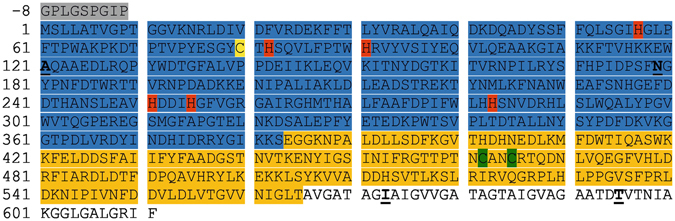
Sequence of the expressed *Ab*PPO4. shaded in grey: vector-derived sequence; blue: central domain up to serine 383, containing the copper-coordinating histidines (red); yellow: cysteine 80 which forms a thioetherbridge with histidine 82; orange: C-terminal domain up to T565 (C-terminus of the main expressed construct); green: CXXC-motif containing all the free cysteine residues of the enzyme; The four amino acid mutations relative to C7FF05 (Table 1) are underlined and marked in **bold**.

ESI-MS of the acidified (causes the loss of copper)^27^ but otherwise intact protein (see Fig. 4) yielded a mass of 65096.7 ± 0.30 Da for the latent tyrosinase which matches the calculated mass of 65096.46 Da for the complete sequence (see Fig. 5) from the N-terminal glycine at position −8 to the C-terminal threonine 565 with one thioether bridge (−2.016 Da) and one closed disulfide bridge (−2.016 Da). The presence of a closed disulfide bridge was also reported for the enzyme isolated from the natural source after electrospray ionisation in positive mode^27^ but the disulfide bridge was found in the open form in the crystal structure^33^. The only free cysteine residues in the protein are very close to each other in the C-terminal domain of the enzyme (C462 and C465). Since positive electrospray ionisation of free cysteine has been shown to generate mainly protonated cystine (which was attributed to oxidation processes occurring during positive mode electrospray)^34^ a similar mechanism may also be observed for the pseudo molecular ions of *Ab*PPO4. The mass spectra of the activated tyrosinase indicated the presence of two protein variants with determined masses of 44181.5 ± 0.51 Da and 44449.6 ± 0.48 Da, respectively. These masses indicate proteolytic cleavage after lysine 382 (calculated: 44449.35 Da) and additionally for most of the tyrosinase molecules the removal of the first three N-terminal amino acids (Gly-Pro-Leu) from the vector-derived region (calculated: 44182.03 Da).

### Crystal structure of latent *Ab*PPO4

**Figure 6:**
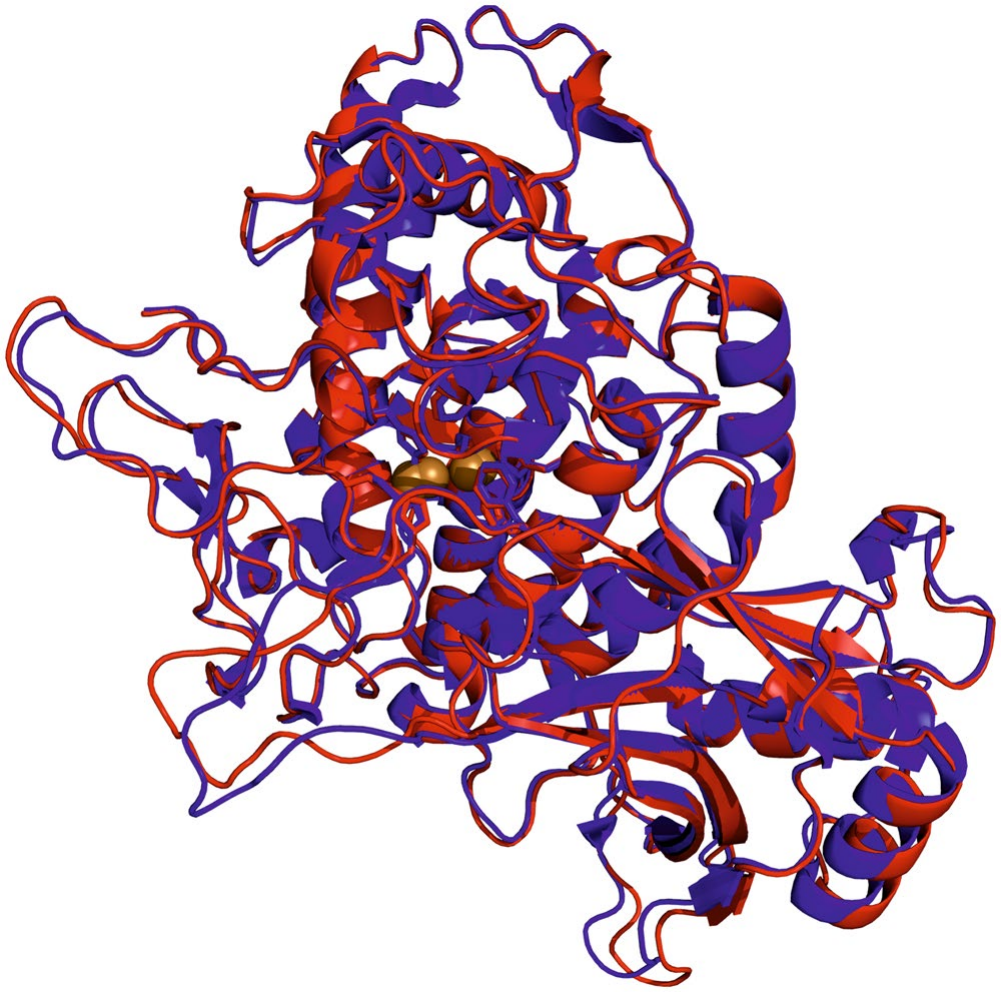
Alignment of *Ab*PPO4 isolated from the natural source and the recombinant enzyme. The latent chain of *Ab*PPO4 isolated from *Agaricus bisporus* (PDB 4OUA, chain B; shown in blue)^36^ was aligned with the heterologously produced protein (PDB 5M6B, chain B; shown in red). The r.m.s.d. between the positions of 3616 matched atoms amounts to 0.435 Å.

Latent tyrosinase crystallized in the monoclinic space group C 1 2 1 with 4 chains in the asymmetric unit giving rise to a unit cell of *a* = 287.30 Å, *b* = 52.09 Å, *c* = 152.66 Å, α = 90.00°, β = 98.03° and γ = 90.00°. The obtained crystals diffracted to a resolution of 3.25 Å (for further statistics see Table S2). In contrast to the crystals obtained with the enzyme isolated from the natural source which contained two different chains in the asymmetric unit (one latent and one active protein)^33^ and did only form in the presence of sodium hexatungstotellurate(VI) (Na_6_[TeW_6_O_24_] • 22 H_2_O, TEW)^35, 36^, the recombinant enzyme formed crystals containing exclusively the latent tyrosinase. Inspection of the electron density gave no indication for any deviation from the sequence shown in Fig. 5. The recombinant enzyme assumes the same fold as the tyrosinase isolated from the natural source and their active centers as well as the surrounding amino acids are virtually identical (see Fig. 6).

The amino acid side chains which form hydrogen bonds with the TEW polyoxyanion in the crystal of the enzyme purified from the natural source (HKKE starting at H116) are found in equivalent positions in the crystal of the recombinant enzyme but the lysine side chains are considerably more flexible as indicated by the lack of electron density beyond the C_β_-atoms. In the crystal containing TEW the interactions mediated by TEW are crucial for the stacking of the monomer chains while in the crystal of recombinant latent *Ab*PPO4 this motif does not play a significant role for the formation of the lattice with the closest interchain contact being longer than 6 Å. The missing contribution from these strong interactions may be one decisive factor limiting the attainable crystallographic resolution^35, 37^ which is 0.49 Å worse than in the crystal structure obtained using TEW.

### Characterization of the activated *Ab*PPO4

The optimal pH for the enzymatic conversion of *L*-tyrosine was found to be 6.8 (Figure S5). Enzymatic activity is found starting from pH 4 and extends beyond pH 10.5. In the pH-range from 5 to 10 the enzyme retains more than 50% of its activity at the optimal pH. The activated *Ab*PPO4 catalyzes both reactions observed for tyrosinase (Fig. 1). The catechol oxidase activity proceeds typically with a rate two orders of magnitude faster than the hydroxylation and oxidation of monophenols (Table 2 and Figure S7). Of the tested substrates the enzyme exhibits the highest affinity and the lowest reaction rate for *L*-tyrosine. The reaction with catechol shows a decrease in rate for catechol concentrations higher than 10 mM which is probably due to the effective suicide-inactivation of tyrosinase by this substrate^38, 39^. Evidence for such a loss of activity during catalysis has also been presented for the type III copper enzyme aurone synthase from Coreopsis grandiflora *Coreopsis grandiflora* acting on sulfuretin (which does contain a catechol moiety)^40^.

**Table 2:** Kinetic parameters of activated *Ab*PPO4.

Substrate	λ_max_/nm	ε_max_/M^−1^ cm^−1^	K_m_/mM	k_cat_/s^−1^
*L*-Tyrosine	507^#^	32900 ± 1600^#^	3.14 ± 0.33	3.77 ± 0.36
*L*-DOPA			26.1 ± 1.1	1810 ± 110
Tyramine	506^#^	33300 ± 1100^#^	9.53 ± 0.71	15.6 ± 0.96
Dopamine			15.2 ± 1.6	2540 ± 130
Phenol	504	36800 ± 2100	12.5 ± 0.41	11.3 ± 0.71
Catechol			4.65 ± 0.28	1080 ± 69

^#^Values taken from^70^; The entries are given as value ± standard deviation.

Besides the substrates chosen for kinetic measurements *Ab*PPO4 was also tested with and does accept tyrosol, chlorogenic acid, p-coumaric acid, 4-*tert*-butlycatechol, octopamine, 4-methylcatechol, resorcinol, hydroquinone, protocatechuic acid, 3,4-dihydroxyphenylacetic acid, pyrogallol and 3-methoxyphenol as well as the flavonols fisetin, quercetin, its glycoside rutin, the flavanone naringenin and the chalcons isoliquiritigenin and butein (see Figure S6 and Figure S8 for structural formulas of substrates accepted by *Ab*PPO4).

Activated *Ab*PPO4 discriminates between enantiomers of tyrosine showing pronounced differences in the rate of the tyrosinase reaction. For tyrosine 1 mM of the *L*-enantiomer is converted at a rate of 1.22 ± 0.0073 U mg^−1^, which is 2.58 ± 0.020 times faster than the rate on *D*-tyrosine. A slight increase in enantioselectivity is seen for the methyl ester of tyrosine for which the respective value is 14.3 ± 0.11 U mg^−1^ for *L*-tyrosine methyl ester representing a ratio of 3.70 ± 0.050 relative to the rate for the *D*-enantiomer.

## Discussion

For bacterial tyrosinases productivities in the gram per liter range have been demonstrated with the application of an optimized fed-batch strategy^41^ but for eukaryotic tyrosinases a yield of 4 to 6 mg per liter of culture is already considered large-scale^42^ and the same value was also reported for a related plant enzyme^43^. Expression strategies targeting fungal tyrosinases did usually rely on fungal hosts for the expression of soluble and active tyrosinase^44–46^. Substantial expression yields per liter of culture were reported for the secreted TYR2 from *Trichoderma reesei* overexpressed homologously (1 g l^−1^ in batch fermentation)^45^ or in *Komagataella pastoris* (24 mg l^−1^)^46^ and for a tyrosinase from *Pycnoporus sanguineus* produced heterologously in *Aspergillus niger* (20 mg l^−1^)^44^. As fungi do possess the necessary molecular tools to activate latent tyrosinases all the isolated enzymes were in their active form^47^. Bacterial expression of fungal tyrosinase does provide access to latent tyrosinases but was hampered by insufficient solubility of the expressed proteins which were also prone to enzymatic inactivity^28^. Enzymatically functional protyrosinase from *Pholiota microspora* was expressed in *Escherichia coli* ^48^ but no value for the yield was reported. Recently, protyrosinase from *Polyporus arcularius* was produced in the same host with a yield of 54 mg latent tyrosinase per liter of culture medium^49^. Here, the latent form of *Ab*PPO4 is produced by *E. coli* at a yield of 110 to 140 mg per liter of culture. The conversion of latent tyrosinase into the enzymatically active form is coupled to the proteolytic removal of the C-terminal domain which shields the active site of the tyrosinase^47, 50^. The causal agents for this key-step in the maturation of fungal tyrosinases are still elusive and for only four fungal tyrosinases the exact location of this crucial event is known. *Neurospora crassa* TYR is activated *in vivo* by cleavage after F408^51^, active TYR2 of *Trichoderma reesei* extends up to G400^45^, TYR1 from *Pholiota nameko* is cleaved after F387^52^ and the C-terminal residue of active *Ab*PPO4 is S383^27^. All those cleavage sites are found 31 or 30 (*T. reesei* TYR2) amino acids after the tyrosine motif (Y-X-Y/F or Y/F-X-Y) which is found close to the end of the central domain in all tyrosinases^53^. Preceding the cleavage site by 4 amino acids, the YG-motif, which is conserved in fungal tyrosinases^20^, is present. The activated *Ab*PPO4 presented herein was cleaved by proteinase K after K382, which is only one amino acid away from the *in vivo* activation site S383^27^. This activated tyrosinase should therefore be an excellent model for the native enzyme. Digestion with proteinase K has also been used as a purification method for tyrosinase from mice^54^ and apple^55^. This stability against proteinase K digestion of tyrosinases from three different kingdoms of life suggests resistance against proteolysis by serine proteases as a general feature of tyrosinases.

Besides proteolytic activation enzymatic activity may also be induced in latent tyrosinases by exposing them to acidic conditions^56^ or detergents like SDS^57^. Employing such a system, the enzymatic activity may be kept dormant in a preparation for a prolonged period of time until it is needed at which point it may be induced by the simple addition of a detergent. Latent *Ab*PPO4 was activated by both the cationic detergent CPC and the anionic detergent SDS (see Fig. 3). Activation by CPC did yield a circa 25% higher activity than SDS-activation did and required only one sixth of the detergent concentration. For routine analysis during purification we employed SDS (at a concentration of 2 mM) as CPC did cause precipitation of preparations still containing significant concentrations of foreign proteins and, at concentrations above 2 mM, also *Ab*PPO4 itself.

Activated *Ab*PPO4 retains >50% of its activity at the optimal pH 6.8 in the range of pH 5 to pH 10 providing a wide range of possible reaction conditions. For most of the characterized tyrosinases this range is considerably more narrow, e.g. for the heterologously expressed tyrosinase from *Polyporus arcularius* it is found between pH 5–6^49^ and the homologously overexpressed *T. reesei* TYR2 was found to be almost fully active in the range of pH 6–9.5^45^.

The kinetic characterization of activated *Ab*PPO4 shows low specificity and high reaction rates for the tested substrates, especially the diphenols (see Table 2). Kinetic parameters of recombinant fungal tyrosinases on *L*-tyrosine were reported for *Ab*PPO2 produced in *Saccharomyces cerevisiae* (K_m_ = 0.302 µM, k_cat_ = 11.39 s^−1^)^58^ and for MelB from *Aspergillus oryzae* produced in *E. coli* (K_m_ = 43 µM, k_cat_ = 49 s^−1^)^59^, making these enzymes both more specific towards *L*-tyrosine as well as faster on this substrate than activated *Ab*PPO4. For TYR1 from *P. nameko* the kinetic parameters on tyrosine could not be determined due to insufficient solubility of the substrate^52^. For *L*-DOPA a slightly higher number of values (enzyme name: K_m_ in µM | k_cat_ in s^−1^) are reported for the recombinant tyrosinases from *A. bisporus* (*Ab*PPO2: 1.22 | 141)^58^, *P. nameko* (TYR1: 1930 | 478)^52^, *P. arcularius* (Photo-regulated tyrosinase: 1040 | 223)^49^ and *T. reesei* (TYR2: 3000 | 22)^45^. In comparison to those enzymes activated *Ab*PPO4 is less specific for *L*-DOPA and much faster on this substrate.

## Conclusions

In conclusion, a protocol for the production of mushroom tyrosinase was established which is able to produce both latent and active tyrosinase in a pure form. Using this protocol it is possible to provide quantities of mushroom tyrosinase sufficient for providing even larger research projects with a defined tyrosinase preparation that does not suffer from the isoenzyme mixture and the side-activities frequently present in commercial preparations of tyrosinase isolated from mushrooms.

## Methods

If not indicated otherwise the chemicals used were purchased from Sigma-Aldrich (Vienna, Austria) or Carl Roth (Karlsruhe, Germany) and were at least of analytical grade. The methyl esters of *L*- and *D*-tyrosine were synthesized at the Department of Biophysical Chemistry, University of Vienna and are characterized by ^1^H-NMR and ESI-MS (see supporting information and Figures S1 and S2).

### RNA-extraction and cDNA-synthesis

RNA extraction and cDNA synthesis have been performed according to standard procedures^60^ and are described in detail in the SI.

### Cloning of the *Ab*PPO4 gene

The gene encoding *Ab*PPO4 was cloned out of frame into the expression vector pGEX-6P-1 (GE Healthcare Europe; Freiburg, Germany) and was brought in frame by PCR-based mutagenesis (for details see SI).

### Mutagenesis of *Ab*PPO4

As the enzyme isolated from the natural source did not contain the last 46 amino acids that are encoded by its gene^27^, the cloned gene was adjusted accordingly. Towards that end the base triplets encoding the respective amino acids were deleted from the gene using the Q5^®^ Site-Directed Mutagenesis Kit (NEB). Removal of the sequence corresponding to amino acids A566 to F611 was accomplished with the two primers l*Ab*PPO4_fwd and l*Ab*PPO4_rev, while E384 to F611 was removed using l*Ab*PPO4_fwd and a*Ab*PPO4_rev resulting in the bases encoding just the main domain of the tyrosinase^27, 33^.

### Expression of *Ab*PPO4

*Ab*PPO4 was expressed in *E. coli* BL21(DE3) in auto-inducing medium, namely ZYM-5052 without the trace element solution^61^ but with an additional 500 mM of NaCl. Cultures were grown at 20 °C in shaking flasks in media containing 100 mg l^−1^ Na-Ampicillin for 20 h. Then, 0.5 mM copper sulfate was added and expression was continued for 20 more hours (for details see SI).

### Isolation and purification of the recombinant tyrosinase

The pelleted cells were washed with 10% of the original culture volume of 9 g l^−1^ NaCl in ddH_2_O, repelleted by centrifugation (8 min @ 3000 × g and 4 °C) and resuspended in lysis buffer (25 mM HEPES, 150 mM NaCl, 5 mM Na_2_MgEDTA set to pH 7.3 with NaOH with the following three components being added immediately before use: 2 mM benzamidine, 1 mM phenylmethylsulfonyl fluoride (PMSF) and 100 mg l^−1^ hen egg white lysozyme) at 100 g of wet cells per l. Cells were disrupted by 2 passages through a french press^62^ at a cell pressure of 850 bar. The samples were cooled on ice in between the passages through the french press and fresh PMSF was added to a final concentration of 2 mM in two portions at the end of each passage. The lysate was cleared by centrifugation (15 min @ 30800 × g and 4 °C), filtered (0.45 µm pore size PES membrane) and applied to a 5 ml GSTrap FF column at a flow rate of 0.5 ml min^−1^. The column was kept at 4 °C and 50 mM Tris-HCl, 150 mM NaCl pH 8.0 (@ 4 °C) was used as the mobile phase for elution of unbound material while the same solution with 20 mM reduced glutathione served as the elution buffer. The eluted fractions containing the fusion protein were concentrated by ultrafiltration (Vivaspin^®^ 20, 30 kDa molecular weight cut-off) which was also applied for buffer exchange.

GST was cleaved from the fusion protein by the action of picornain 3C (human rhinovirus serotype 14 protease 3C, HRV 3C) which was applied as a fusion-protein with GST (production protocol in the SI). 1 µg of protease was applied per 150 µg of GST-*Ab*PPO4 fusion protein and the proteolysis reaction was allowed to proceed for at least 18 h at 4 °C in the running buffer of the affinity chromatography supplemented with 1 mM DTT in order to preserve the activity of the cysteine protease HRV 3C.

After enzymatic cleavage, the tyrosinase was separated from the fusion partner as well as the protease by a second passage through the affinity column using identical conditions as for the first chromatographic step. The column flow-through containing the protein of interest was concentrated by ultrafiltration (Vivaspin® 20, 30 kDa molecular weight cut-off) and its buffer was exchanged to 10 mM HEPES pH 7.5 (@ 4 °C). This preparation was diluted to a concentration of 20 g l^−1^ and stored at 4 °C until use.

### Enzymatic activity assay

Tyrosinase activity was routinely assayed on 1 mM *L*-tyrosine in 50 mM sodium citrate buffer pH 6.8 at 25 °C. For activation of the latent enzyme 2 mM SDS were included in the assay mixture^27^. The monitored species was dopachrome at 475 nm (ε_475_ = 3600 M^−1^ cm^−1^)^63^, volumetric enzymatic activities were calculated from the linear part of the absorption-time curves (after the lag-phase but before the subsequent reactions towards melanin contribute significantly). One unit of enzymatic activity (U) was defined as the amount of enzyme that catalyzes the conversion of 1 µmol of substrate per minute of reaction.

### Intact protein mass spectrometry

For ESI-MS, which was done at the Mass Spectrometry Centre at the University of Vienna, the buffer of the protein solutions was exchanged to 5 mM ammonium hydrogen carbonate pH 7.8 by repeated ultrafiltration (Vivaspin^®^ 500, 30 kDa molecular weight cut-off). For introduction into the nanoESI-QTOF mass spectrometer (maXis 4 G UHR-TOF from Bruker, Billerica, MA, USA; providing a mass accuracy better than 5 ppm) by a syringe pump (KDS 100 from KD Scientific, Holliston, MA, USA) @ 3 µl min^−1^ the protein solutions were diluted to approximately 1 µM in an aqueous solution containing 2% (v/v) acetonitrile and 1‰ (v/v) formic acid.

### Enzyme kinetics

A spectrophotometric assay detecting the appearance of the product in the reaction solution was applied for the determination of the kinetic parameters of *Ab*PPO4. Since the *o*-quinones generated by the enzymatic action of tyrosinase do not give rise to a stable and soluble product they were trapped by the potent nucleophile 3-methyl-2-benzothiazolinone hydrazone (MBTH). MBTH couples to *o*-quinones via its amino group generating reasonably stable adducts that remain soluble and are easily detected photometrically^64^. Absorption curves and spectra were recorded on a Shimadzu UV-1800 spectrophotometer applying 1 cm cuvettes which were kept at 25 °C by a Julabo F25 MH thermostat in a circulating water-bath. Kinetic measurements were done in a total volume of 1 ml containing 50 mM sodium citrate buffer pH 6.8, 5 mM MBTH, 2% (v/v) N,N-dimethylformamide and different concentrations of the substrates to be tested as well as *Ab*PPO4 (0.23 to 46 nM).

Molar absorption coefficients were determined from rapid oxidation of small concentrations of substrate under standard assay conditions applying tyrosinase concentrations in the µM range. K_m_ and k_cat_, the two parameters of the Michaelis-Menten model^65^, were calculated from the steady-state rate of product formation for the different substrate concentrations tested. Measurements were performed in triplicate and the reciprocals of the variances of the observed slopes were used as weights for the nonlinear regression applying the Levenberg-Marquardt algorithm^66^ as implemented in the program Dataplot (version 11/2010). Initial estimates for the two free parameters were generated by applying the Hanes-Woolf linearization of the Michaelis-Menten equation^67^.

### Proteolytic activation of *Ab*PPO4

Latent *Ab*PPO4 was converted into its active form by treatment with proteinase K. The protease was used at a ratio of 1:10 (equivalent to 45 µg of proteinase K per mg of latent *Ab*PPO4) in a reaction buffer containing 50 mM Tris-HCl pH 8 @ 25 °C, 100 mM sodium ascorbate and 20 g l^−1^ of latent *Ab*PPO4 for a total reaction time of 90 min. The reaction was stopped by addition of 2 mM PMSF after which the solution was concentrated to less than 70 µl by ultrafiltration (Vivaspin^®^ 500, 30 kDa molecular weight cut-off) and applied onto a size exclusion column (Superdex 200 Increase from GE Healthcare) equilibrated with 50 mM sodium citrate pH 6.8 and run with the same buffer at 4 °C and with a flow rate of 0.5 ml min^−1^. The eluted fractions possessing tyrosinase activity were pooled and concentrated by ultrafiltration (Vivaspin^®^ 500, 30 kDa molecular weight cut-off).

### Protein crystallization, X-ray diffraction and model building

Conditions for the growth of *Ab*PPO4 crystals were refined based on an initial hit obtained with sodium cacodylate and PEG 4000 in a hanging drop vapour diffusion setup. Single crystals suitable for crystallography grew over the course of a few days in hanging drops initially made up of 1 µl of a 10 g l^−1^ solution of latent *Ab*PPO4 in 10 mM HEPES pH 7.5 mixed with 1 µl of reservoir solution containing 50 mM sodium cacodylate pH 5.8 and 13% (w/w) PEG 4000 which was equilibrated via vapour diffusion with 1 ml of reservoir solution at 293 K. Crystals were harvested using Kapton^®^ loops (Hampton Research, Aliso Viejo, CA, USA), soaked with cryo-protectant (50 mM sodium cacodylate pH 5.8 and 40% (w/w) PEG 4000) and plunged into liquid nitrogen where they remained until the diffraction experiment.

Data were collected at beamline ID-23 at the European Synchrotron Radiation Facility (ESRF, Grenoble, France) at 100 K applying a wavelength of 0.873 Å (14.2 keV) and a PILATUS2 3 M detector. Data reduction (for details see SI) was carried out using XDS^68^. Final model quality was evaluated by the MolProbity server^69^ and the model has been deposited in the PDB under entry number 5M6B.

## Electronic supplementary material

SI

## References

[1] Claus H, Decker H (2006). Bacterial tyrosinases. Syst. Appl. Microbiol..

[2] Lerner AB (1952). Effect of Ions on Melanin Formation. J. Invest. Dermatol..

[3] Mason HS (1948). The Chemistry of Melanin: III. Mechanism of the Oxidation of Dihydroxyphenylalanine by Tyrosinase. J. Biol. Chem..

[4] Rodríguez-López JN, Tudela J, Varón R, García-Carmona F, García-Cánovas F (1992). Analysis of a kinetic model for melanin biosynthesis pathway. J. Biol. Chem..

[5] Pfiffner E, Lerch K (1981). Histidine at the active site of Neurospora tyrosinase. Biochemistry.

[6] Hazes B (1993). Crystal structure of deoxygenated Limulus polyphemus subunit II hemocyanin at 2.18A resolution: clues for a mechanism for allosteric regulation. Protein Sci..

[7] Kitajima N, Fujisawa K, Moro-oka Y, Toriumi K (1989). µ-η^2^:η^2^-Peroxo binuclear copper complex, [Cu(HB(3,5-(Me_2_CH)_2_pz)_3_)]_2_(O_2_). J. Am. Chem. Soc..

[8] Rompel A (1995). Spectroscopic and EXAFS studies on catechol oxidases with dinuclear copper centers of type 3: Evidence for μ-η^2^:η^2^-peroxo-intermediates during the reaction with catechol. J. Inorg. Biochem..

[9] Rompel A (1999). Purification and spectroscopic studies on catechol oxidases from *Lycopus europaeus* and *Populus nigra*: Evidence for a dinuclear copper center of type 3 and spectroscopic similarities to tyrosinase and hemocyanin. JBIC J. Biol. Inorg. Chem..

[10] Ito S (2003). A Chemist’s View of Melanogenesis. Pigment Cell Res.

[11] Hearing VJ, Tsukamoto K (1991). Enzymatic control of pigmentation in mammals. FASEB J..

[12] Mason HS, Wright CI (1949). The chemistry of melanin v. oxidation of dihydroxyphenylalanine by tyrosinase. J. Biol. Chem..

[13] Pawelek JM (1991). After Dopachrome?. Pigment Cell Res..

[14] Polacheck I, Kwon-Chung KJ (1988). Melanogenesis in Cryptococcus neoformans. J. Gen. Microbiol..

[15] Burzio LA, Waite JH (2001). Reactivity of peptidyl-tyrosine to hydroxylation and cross-linking. Protein Sci..

[16] Ito S, Kato T, Shinpo K, Fujita K (1984). Oxidation of tyrosine residues in proteins by tyrosinase. Formation of protein-bonded 3,4-dihydroxyphenylalanine and 5-S-cysteinyl-3,4-dihydroxyphenylalanine. Biochem. J..

[17] Burzio LA, Waite JH (2000). Cross-Linking in Adhesive Quinoproteins: Studies with Model Decapeptides. Biochemistry.

[18] McDowell LM, Burzio LA, Waite JH, Schaefer J (1999). Rotational Echo Double Resonance Detection of Cross-links Formed in Mussel Byssus under High-Flow Stress. J. Biol. Chem..

[19] Faccio G, Kruus K, Saloheimo M, Thöny-Meyer L (2012). Bacterial tyrosinases and their applications. Process Biochem..

[20] Pretzler, M., Bijelic, A., Rompel, A. Fungal Tyrosinases: Why Mushrooms Turn Brown. In: *Ref. Module Chem. Mol. Sci. Chem. Eng*. Elsevier, doi:10.1016/B978-0-12-409547-2.11521-5 (2015).

[21] Seo S-Y, Sharma VK, Sharma N (2003). Mushroom Tyrosinase: Recent Prospects. J. Agric. Food Chem..

[22] Flurkey A (2008). Enzyme, Protein, Carbohydrate, and Phenolic Contaminants in Commercial Tyrosinase Preparations: Potential Problems Affecting Tyrosinase Activity and Inhibition Studies. J. Agric. Food Chem..

[23] Rescigno A, Zucca P, Flurkey A, Inlow J, Flurkey WH (2007). Identification and discrimination between some contaminant enzyme activities in commercial preparations of mushroom tyrosinase. Enzyme Microb. Technol..

[24] Morin E (2012). Genome sequence of the button mushroom *Agaricus bisporus* reveals mechanisms governing adaptation to a humic-rich ecological niche. Proc. Natl. Acad. Sci.

[25] Weijn A, Bastiaan-Net S, Wichers HJ, Mes JJ (2013). Melanin biosynthesis pathway in *Agaricus bisporus* mushrooms. Fungal Genet. Biol..

[26] Ismaya WT (2011). Crystal Structure of Agaricus bisporus Mushroom Tyrosinase: Identity of the Tetramer Subunits and Interaction with Tropolone. Biochemistry.

[27] Mauracher SG (2014). High level protein-purification allows the unambiguous polypeptide determination of latent isoform PPO4 of mushroom tyrosinase. Phytochemistry.

[28] Wu J (2010). Cloning, characterization and expression of two new polyphenol oxidase cDNAs from *Agaricus bisporus*. Biotechnol. Lett..

[29] Kumar P, Henikoff S, Ng PC (2009). Predicting the effects of coding non-synonymous variants on protein function using the SIFT algorithm. Nat. Protoc..

[30] Smith DB, Johnson KS (1988). Single-step purification of polypeptides expressed in *Escherichia coli* as fusions with glutathione S-transferase. Gene.

[31] Dirks-Hofmeister ME, Kolkenbrock S, Moerschbacher BM (2013). Parameters That Enhance the Bacterial Expression of Active Plant Polyphenol Oxidases. PLoS ONE.

[32] Deng J (2004). An improved protocol for rapid freezing of protein samples for long-term storage. Acta Crystallogr. Sect. D.

[33] Mauracher SG, Molitor C, Al-Oweini R, Kortz U, Rompel A (2014). Latent and active *ab*PPO4 mushroom tyrosinase cocrystallized with hexatungstotellurate(VI) in a single crystal. Acta Crystallogr. Sect. D.

[34] Steill JD, Szczepanski J, Oomens J, Eyler JR, Brajter-Toth A (2011). Structural characterization by infrared multiple photon dissociation spectroscopy of protonated gas-phase ions obtained by electrospray ionization of cysteine and dopamine. Anal. Bioanal. Chem..

[35] Bijelic A, Rompel A (2015). The use of polyoxometalates in protein crystallography–An attempt to widen a well-known bottleneck. Coord. Chem. Rev..

[36] Mauracher SG, Molitor C, Al-Oweini R, Kortz U, Rompel A (2014). Crystallization and preliminary X-ray crystallographic analysis of latent isoform PPO4 mushroom (*Agaricus bisporus*) tyrosinase. Acta Crystallogr. Sect. F.

[37] Molitor C, Bijelic A, Rompel A (2016). *In situ* formation of the first proteinogenically functionalized [TeW_6_O_24_O_2_(Glu)]^7−^ structure reveals unprecedented chemical and geometrical features of the Anderson-type cluster. Chem. Commun..

[38] Land EJ, Ramsden CA, Riley PA (2007). The Mechanism of Suicide-Inactivation of Tyrosinase: A Substrate Structure Investigation. Tohoku J. Exp. Med..

[39] Muñoz-Muñoz JL (2012). Unravelling the suicide inactivation of tyrosinase: A discrimination between mechanisms. J. Mol. Catal. B Enzym.

[40] Molitor C (2015). Latent and active aurone synthase from petals of *C. grandiflora*: a polyphenol oxidase with unique characteristics. Planta.

[41] Ren Q, Henes B, Fairhead M, Thony-Meyer L (2013). High level production of tyrosinase in recombinant Escherichia coli. BMC Biotechnol..

[42] Lai X, Soler-Lopez M, Wichers HJ, Dijkstra BW (2016). Large-Scale Recombinant Expression and Purification of Human Tyrosinase Suitable for Structural Studies. PLoS ONE.

[43] Kaintz C (2014). Cloning and functional expression in E. coli of a polyphenol oxidase transcript from *Coreopsis grandiflora* involved in aurone formation. FEBS Lett.

[44] Halaouli S (2006). Cloning and characterization of a tyrosinase gene from the white-rot fungus *Pycnoporus sanguineus*, and overproduction of the recombinant protein in *Aspergillus niger*. Appl. Microbiol. Biotechnol..

[45] Selinheimo E (2006). Production and characterization of a secreted, C-terminally processed tyrosinase from the filamentous fungus *Trichoderma reesei*. FEBS J..

[46] Westerholm-Parvinen A (2007). Expression of the Trichoderma reesei tyrosinase 2 in Pichia pastoris: Isotopic labeling and physicochemical characterization. Protein Expr. Purif..

[47] Faccio G, Arvas M, Thöny-Meyer L, Saloheimo M (2013). Experimental and bioinformatic investigation of the proteolytic degradation of the C-terminal domain of a fungal tyrosinase. J. Inorg. Biochem..

[48] Kawamura-Konishi Y, Maekawa S, Tsuji M, Goto H (2011). C-terminal processing of tyrosinase is responsible for activation of *Pholiota microspora* proenzyme. Appl. Microbiol. Biotechnol..

[49] Marková E (2016). Recombinant Tyrosinase from *Polyporus arcularius*: Overproduction in *Escherichia coli*, Characterization and Use in a Study of Aurones as Tyrosinase Effectors. J. Agric. Food Chem..

[50] Flurkey WH, Inlow JK (2008). Proteolytic processing of polyphenol oxidase from plants and fungi. J. Inorg. Biochem..

[51] Lerch K (1978). Amino acid sequence of tyrosinase from *Neurospora crassa*. Proc. Natl. Acad. Sci..

[52] Kawamura-Konishi Y (2007). Purification, Characterization, and Molecular Cloning of Tyrosinase from Pholiota nameko.. Biosci. Biotechnol. Biochem..

[53] Marusek CM, Trobaugh NM, Flurkey WH, Inlow JK (2006). Comparative analysis of polyphenol oxidase from plant and fungal species. J. Inorg. Biochem..

[54] Yurkow EJ, Laskin JD (1989). Purification of tyrosinase to homogeneity based on its resistance to sodium dodecyl sulfate-proteinase K digestion. Arch. Biochem. Biophys..

[55] Marqués L, Fleuriet A, Cleyet-Marel J-C, Macheix J-J (1994). Purification of an apple polyphenol oxidase isoform resistant to SDS-proteinase K digestion. Phytochemistry.

[56] Fujita Y, Uraga Y, Ichisima E (1995). Molecular cloning and nucleotide sequence of the protyrosinase gene, melO, from *Aspergillus oryzae* and expression of the gene in yeast cells. *Biochim. Biophys. Acta BBA*. Gene Struct. Expr.

[57] Espín JC, Wichers HJ (1999). Activation of a Latent Mushroom (*Agaricus bisporus*) Tyrosinase Isoform by Sodium Dodecyl Sulfate (SDS). Kinetic Properties of the SDS-Activated Isoform. J. Agric. Food Chem..

[58] Lezzi C, Bleve G, Spagnolo S, Perrotta C, Grieco F (2012). Production of recombinant Agaricus bisporus tyrosinase in Saccharomyces cerevisiae cells. J. Ind. Microbiol. Biotechnol..

[59] Fujieda N (2012). Multifunctions of MelB, a Fungal Tyrosinase from Aspergillus oryzae. ChemBioChem.

[60] Mülhardt, C. Der Experimentator Molekularbiologie/Genomics. In: Mülhardt, C., editor. Springer Spektrum. Experimentator 7, pp. 140–142 (2013).

[61] Studier FW (2005). Protein production by auto-induction in high-density shaking cultures. Protein Expr. Purif..

[62] French, C. S., Milner, H. W. [9] Disintegration of bacteria and small particles by high-pressure extrusion. In: Academic Press. Methods in Enzymology, Vol. 1, pp. 64–67 (1955).

[63] Gandía-Herrero F, Jiménez-Atiénzar M, Cabanes J, García-Carmona F, Escribano J (2005). Differential Activation of a Latent Polyphenol Oxidase Mediated by Sodium Dodecyl Sulfate. J. Agric. Food Chem..

[64] Winder AJ, Harris H (1991). New assays for the tyrosine hydroxylase and dopa oxidase activities of tyrosinase. Eur. J. Biochem..

[65] Michaelis L, Menten ML (1913). Die Kinetik der Invertinwirkung. Biochem. Z..

[66] Marquardt D (1963). An Algorithm for Least-Squares Estimation of Nonlinear Parameters. J. Soc. Ind. Appl. Math..

[67] Hanes CS (1932). CLXVII. Studies on plant amylases I. The effect of starch concentration upon the velocity of hydrolysis by the amylase of germinated barley. Biochem. J..

[68] Kabsch W (2010). XDS. Acta Crystallogr. Sect. D.

[69] Chen VB (2010). MolProbity: all-atom structure validation for macromolecular crystallography. Acta Crystallogr. Sect. D.

[70] Bijelic, A., Pretzler, M., Molitor, C., Zekiri, F. & Rompel, A. The Structure of a Plant Tyrosinase from Walnut Leaves Reveals the Importance of “Substrate-Guiding Residues” for Enzymatic Specificity. *Angew. Chem. Int. Ed*. **54**, 14677–14680, doi:10.1002/anie.201506994 (2015); *Angew. Chem*. **127**, 14889–14893, doi:10.1002/ange.201506994 (2015). 10.1002/anie.201506994 PMC4678486 26473311

