# Supplementary material for: Heterologous expression and characterization of functional mushroom tyrosinase (AbPPO4)
Source: Sci Rep. 2017 May 12;7:1810. doi: 10.1038/s41598-017-01813-1 (PMC5431950; doi:10.1038/s41598-017-01813-1)
Supplement: Supplementary file 1 — SI [file 41598_2017_1813_MOESM1_ESM.pdf]

Supplementary Information

Heterologous expression and characterization of functional mushroom tyrosinase (*Ab*PPO4)

Matthias Pretzler[1], Aleksandar Bijelic[1] & Annette Rompel[1]*

[1] Universität Wien, Fakultät für Chemie, Institut für Biophysikalische Chemie, Althanstraße 14, 1090 Wien, Austria; http://www.bpc.univie.ac.at

*Correspondence to annette.rompel@univie.ac.at

Methods

RNA-extraction and cDNA-synthesis. Total RNA was extracted from a 1.20 g (wet, dry weight was approximately 9 %) piece of the stipe taken from an *Agaricus bisporus* fruiting body (hybrid strain Horst U1) at developmental stage 5.[1,2] Extraction was carried out using the RNeasy® Plant Mini Kit from QIAGEN (Hilden, Germany) according to the manufacturer's instructions with a lysis buffer containing 4 M guanidine isothiocyanate and 1 % (v/v) β-mercaptoethanol. The resulting RNA was used as template for the synthesis of cDNA by SMARTScribe™ Reverse Transcriptase (Clontech; Mountain View, CA, USA) with an oligo(dT)-primer (5'-T_{25}VN -3') following the protocol recommended by the manufacturer.

Cloning of the *Ab*PPO4 gene. The gene encoding *Ab*PPO4 was amplified from the *A. bisporus* cDNA by PCR with the Q5® High-Fidelity DNA polymerase (NEB: New England Biolabs; Ipswich, MA, USA). PCR was done in a total volume of 20 µl containing 500 nM of both primers *Ab*PPO4_fwd and *Ab*PPO4_rev (The sequence of all used primers may be found in Table S1.), 200 µM of each of the four dNTPs, 2 µl of the cDNA preparation, 1-fold Q5® Reaction Buffer (contains 2 mM $MgCl_2$) and 0.4 units of Q5® DNA polymerase. Cycling was done in a Mastercycler® pro (Eppendorf; Hamburg, Germany) set to the following program: initial denaturation for 30 s @ 98 °C followed by 35 cycles of 12 s @ 98 °C, 40 s @ 66 °C and 70 s @ 72 °C and an final elongation for 7 min @ 72 °C. The reaction yielded one main band at circa 1.8 kbp and a much fainter one around 1.4 kbp. Exploratory PCR with *Taq* DNA polymerase on the *A. bisporus* cDNA applying the same primers did also persistently yield two amplicons of approximately 1.8 kbp and 1.4 kbp for all annealing temperatures tested (45 °C to 65 °C, Figure S3).

The amplicons from the reaction with Q5® were purified by agarose gel electrophoresis followed by a silica membrane based DNA recovery step (Wizard® SV Gel and PCR Clean-Up System from Promega; Madison, WI, USA).

The amplicon was cloned out-of-frame into the expression vector pGEX-6P-1 (GE Healthcare Europe; Freiburg, Germany) linearized by digestion with *Sma*I (NEB) via cut-ligation.[3] Briefly, 30 fmol of pGEX-6P-1 cut with *Sma*I were incubated with 90 fmol of the purified amplicon, 1 mM ATP, 4 units of *Sma*I and 400 cohesive end ligation units of T4 DNA ligase (NEB) in a total volume of 20 µl 1 x CutSmart® buffer (NEB: 50 mM potassium acetate, 20 mM Tris-acetate, 10 mM magnesium acetate,

0.1 g l^{-1} BSA; pH 7.9 @ 25°C) for 16 h @ 16 °C followed by thermal denaturation of the enzymes by heating to 65 °C for 10 min. Before transformation the ligation mixture was incubated with an additional 10 units of *Sma*I for 2 h @ 25 °C in order to suppress background colonies arising from transformation of religated vector.

Chemically competent cells (*E. coli* NEB 5-alpha) were prepared according to the $CaCl_2$-method[4] and stored frozen at -80 °C. 6 µl of the ligation mixture (after additional digestion with *Sma*I) were transformed into NEB 5-alpha cells using the heat shock protocol.[5] Transformants were selected on LB agar plates with 100 mg l^{-1} Na-Ampicillin by incubation at 37 °C overnight. The colonies that formed on this selective media were tested for the presence of the *Ab*PPO4-gene in the right orientation by colony PCR[6] with *Taq* DNA polymerase (Hot Start *Taq* DNA polymerase from NEB) and the primers pGEX_fwd and *Ab*PPO4_rev. Briefly, 10 µl reactions were set up containing 500 nM of each primer, 5 µl Hot Start *Taq* 2X Master Mix (final concentrations: 10 mM Tris-HCl, 50 mM KCl, 1.5 mM $MgCl_2$, 200 µM of each dNTP, 5 % (v/v) glycerol, 25 units ml^{-1} Hot Start *Taq* DNA polymerase; pH 8.6 @ 25°C), ddH_2O to 10 µl and a few nl from the bacterial colony to be tested. PCR was carried out as follows: 7 min @ 95 °C for cell lysis and initial denaturation of the DNA, 35 cycles of 40 s @ 94 °C, 60 s @ 59 °C, 140 s @ 72 °C, where the annealing temperature (59 °C) was initially set to 67 °C and was lowered by 1 °C per cycle for the first 9 cycles, and 10 min @ 72 °C for the final elongation. Positive clones were taken into liquid culture (12 ml SB with 100 mg l^{-1} Na-Ampicillin, incubation overnight at 37 °C and 250 min^{-1}) which were subsequently used for plasmid DNA extraction (Wizard® Plus SV Minipreps DNA Purification System, Promega). The purified plasmids were analyzed in the region of the insert by Sanger sequencing of both DNA strands (Microsynth; Vienna, Austria).

In order to bring the *Ab*PPO4-gene in frame with the fusion partner glutathione S-transferase (GST) encoded by the pGEX vector a single deoxyadenosine monophosphate was inserted in the region before the open reading frame of the gene for *Ab*PPO4. This was done applying the Q5® Site-Directed Mutagenesis Kit (NEB) according to the manufacturers recommendations with the two primers pGEX-6P-1:2_*Ab*PPO4_fwd and pGEX-6P-1:2_rev resulting in a primer annealing temperature of 72 °C. The resulting DNA constructs were transformed into *E. coli* NEB 5-alpha and analyzed by colony PCR and Sanger sequencing as described for the initial cloning step.

Expression of *Ab*PPO4. Sequence-verified plasmids were used to transform chemically competent *E. coli* BL21(DE3). The resulting transformants were selected on LB with 100 mg l^{-1} Na-Ampicillin and positive transformants were tested for the presence of the expression vector by colony PCR as described for the cloning of the gene for *Ab*PPO4. One transformant per variant of the expression plasmid was grown in 10 ml of liquid medium (LB with 2 mM $MgSO_4$ and 100 mg l^{-1} Na-Ampicillin) overnight @ 37 °C and 250 min^{-1}. These cultures were then concentrated to 1 ml by centrifugation at 1000 x g for 2 min @ 30 °C, mixed with an equal volume of 50 % (v/v) glycerol and snap-frozen in liquid nitrogen. Those cryostocks were stored at -80 °C and served as an invariant source of inoculum for the subsequent expression cultures.

Auto-inducing medium was applied for the expression, namely ZYM-5052[7] without the trace element solution and with added Cu^{2+} to increase the activity of the heterologous enzyme.[8] Starter cultures were grown in a 300 ml Erlenmeyer flask in 150 ml of LB medium supplemented with 2 mM $MgSO_4$, 500 mM NaCl and 100 mg l^{-1} Na-Ampicillin. These cultures were incubated overnight at 37 °C and 240 min^{-1} and did function as the source of the inoculum for the main expression cultures. Expression was done in 1000 ml Erlenmeyer flasks filled with 500 ml of autoinducing media comprising LB medium supplemented with 2 mM $MgSO_4$, 500 mM NaCl, 1X mineral stock M (50X stock solution: 1.25 M Na_2HPO_4, 1.25 M KH_2PO_4, 2.5 M NH_4Cl, 0.25 M Na_2SO_4; the solution was heated to approximately 50 °C in order to dissolve all the salts and was then sterile filtrated.)[7], 1X sugar stock 5052 (50X stock solution: 250 g l^{-1} glycerol, 25 g l^{-1} D-glucose, 100 g l^{-1} α-D-lactose monohydrate; the solution was sterile filtrated.)[7] and 100 mg l^{-1} Na-Ampicillin. The main cultures were inoculated to an initial OD_{600} of 0.02 cm^{-1} and were grown at 20 °C and 185 min^{-1} until the OD_{600} reached approximately 2 cm^{-1} which usually took around 20 h. At this point $CuSO_4$ was added to a final concentration of 0.5 mM. Addition of copper was delayed to avoid untimely depletion of the β-lactam antibiotic as Cu(II) promotes the hydrolysis of these compounds.[9] With all the components in place expression was allowed to continue for approximately 20 more hours after which the OD_{600} of the expression cultures was close to 7 cm^{-1} at which point the cultures were harvested by centrifugation (8 min @ 3000 x g and 4 °C).

Expression and purification of HRV 3C. The gene for HRV 3C (K02121.1|5240-5786, with an added stop codon 3' of the sequence coding for the protease) was synthesised by Eurofins Genomics (Ebersberg, Germany) and was transferred from the plasmid pEX-A2 to pGEX-6P-1 using restriction endonuclease recognition sequences added to the gene sequence (5': *BamH*I, 3': *Xho*I). The resulting construct did contain an recognition sequence for HRV 3C (LEVLFQ↑GP) which was replaced by the enterokinase recognition (DDDDK↑) sequence using the Q5® Site-Directed Mutagenesis Kit (NEB) with the primers 3C->EK_for and 3C->EK_rev.

The resulting plasmid was transformed into chemically competent *E. coli* BL21 which served as the expression strain. Expression was done in ZYM-5052[7] autoinducing medium following the protocol devised by Dr. Arie Geerlof (Helmholtz Zentrum München); precultures were grown overnight at 37 °C in SB-media supplemented with 100 mg l^{-1} of Na-Ampicillin. Those cultures were used to inoculate 1 l of ZYM-5052 medium (with 100 mg l^{-1} Na-Ampicillin) in 2 l Erlenmeyer flasks which were subsequently cultivated at 37 °C until the OD_{600} reached circa 1 cm^{-1} at which point the cultivation temperature was lowered to 20 °C. After 16 h of cultivation at 20 °C ($OD_{600} \approx 4\ cm^{-1}$) the cells were harvested by centrifugation (10 min @ 7000 x g and 20 °C), washed with 45 ml of 9 g l^{-1} NaCl and resuspended at 150 g wet cells per l in lysis buffer (50 mM Tris-HCl, 300 m NaCl, 10 %(v/v) glycerol, 0,2% (v/v) Igepal CA-630, 2.5 mM DTT, 0.5 g l^{-1} hen egg white lysozyme).

Cells were broken by two passages through a french press[10] at a cell pressure of 975 bar. The resulting lysate was cleared by centrifugation (90 min @ 3200 x g and 4 °C), filtered (0.45 µM PES-membrane) and applied to a column packed with 8 ml of Glutathion-Sepharose 4B which was operated at 4 °C. The mobile phase used to wash out unbound material consisted of 50 mM Tris-HCl, 300 mM NaCl, 10 % (v/v) glycerol and 1.25 mM DTT with a pH of 8.0 @ 4 °C while the same solution supplemented with 20 mM reduced glutathione was applied for elution of the GST-fusion protein. The eluted fractions containing the GST-picornain 3C fusion protein were concentrated by ultrafiltration (Vivaspin® 20, 30 kDa molecular weigth cut-off) and the buffer was exchanged to the storage buffer (50 mM Tris-HCl, 150 mM NaCl, 10 mM EDTA, 1.25 mM DTT, 20 % (v/v) glycerol; pH 8.0 @ 4°C). The purified fusion protein (yield: 60 mg per liter of autoinduction medium) was aliquoted at 5 g l^{-1}, snap-frozen in liquid nitrogen and stored at -80 °C.

Protein crystallography: model building. Initial phases were obtained by molecular replacement using PHASER[11] supplied with structure factors derived from the crystal structure of *Ab*PPO4 purified from the natural source (PDB 4OUA, chain B).[12] For solving the structure programs from both the CCP4[13] and PHENIX[14] suites were applied. Refinement and rebuilding was performed by employing PHENIX (phenix.refine) and Coot.[15] In order to improve the model and reduce model bias, a first refinement step was carried out in which the atoms were "shaken" by displacing them using a r.m.s.d. distance of 0.8 Å.[16] The resulting partially randomized model was then subjected to simulated annealing using torsion angle dynamic to minimize model bias as much as possible.

Synthesis of tyrosine methyl esters. *L*-tyrosine (0.9 g, 5 mmol) was dissolved in methanol and thionyl chloride (0.545 ml, 7.5 mmol) was added slowly at 0 °C. After refluxing for 2 h, the reaction mixture was poured into ice water (20 ml), neutralized with $NaHCO_3$ and extracted with EtOAc (2 x 20 ml). The organic extracts were combined and dried over $MgSO_4$. The evaporation of the organic solvent lead to 0.550 g (= 2.82 mmol, 56 %) of *L*-tyrosine methyl ester.

D-tyrosine methyl ester: The synthesis was similar to that of *L*-tyrosine methyl ester, except that *D*-tyrosine was used instead of *L*-tyrosine.

Nuclear magnetic resonance: NMR spectra were recorded on a Bruker (Billerica, MA, USA) FT-NMR Avance III 500 MHz instrument at 500.32 (^{1}H) MHz in D_2O at ambient temperatures. Chemical shifts were referenced relative to the solvent signal for ^{1}H. The splitting of proton resonances in the ^{1}H NMR spectra are defined as s = singlet, bs = broad singlet, d = doublet, dd = doublet of doublets, ddd = doublet of doublets of doublets, t = triplet, dt = doublet of triplets, and m = multiplets.

Signal assignment **^{1}H NMR** (500.32 MHz, [D6] DMSO, 25 °C, TMS): δ = 2.7 (m, 2 H), δ = 3.48 (t, 1 H), δ = 3.56 (s, 3 H), δ = 6.64-6.94 (dd, 4 H) ppm for *L*-tyrosine methyl ester. The signals for *D*-tyrosine methyl ester are the same.

other signals: 2.5 ppm: DMSO, 3.34 ppm: methanol, 9.18 ppm: HOOC- from residual tyrosine

Electrospray ionisation mass spectrometry: ESI-MS spectra were taken on a maXis 4G UHR-TOF from Bruker in positive ionisation mode (4.5 kV potential

difference, APCI heater set to 400 °C) at a nebulizer pressure of 0.4 bar and 4 l min^{-1} of dry gas (180 °C) using the TOF-analyser in reflectron mode (mass accuracy of 5 ppm). The samples were dissolved in a 1:1 mixture of acetonitrile and methanol with 1 % (v/v) water and injected at 3 µl min^{-1}.

L-tyrosine methyl ester: 196.0972 m/z (calculated for $[M+H]^+$: 196.0974 m/z)

D-tyrosine methyl ester: 196.0972 m/z (calculated for $[M+H]^+$: 196.0974 m/z)

isotopic distribution

peak area in % of the monoisotopic peak	+1	+2
theoretical ($C_{10}H_{14}NO_3^+$):	11.51	1.22
L-tyrosine methyl ester:	12.32	1.41
D-tyrosine methyl ester:	11.45	1.20

assignment of minor peaks present in the spectrums of both *L*- and *D*-tyrosine methyl ester (given as m/z values): 413.1691 $[2M + Na]^+$, 391.1868 $[2M + H]^+$, 218.0791 $[M + Na]^+$, 179.0707 $[M + H - NH_3]^+$, 151.0760 $[M + H - NH_3 - CO]^+$, 147.0443 $[M + H - NH_3 - MeOH]^+$, 136.0759 $[M + H - C_2H_4O_2]^+$, 119.0495 $[M + H - C_2H_4O_2 - NH_3]^+$

Tables

Table S1: List of primers used

Designation	Sequence (5' - 3')	Use
*Ab*PPO4_fwd	*ATG*TCTCTGCTCGCTACTG	Amplification of *ab*PPO4 from *A. bisporus* cDNA
*Ab*PPO4_rev	*TTA*AAAGATACGTCCCAAAGCAC	Amplification of *ab*PPO4 from *A. bisporus* cDNA
pGEX_fwd	TGTCCAAAAGAGCGTGCAGA	Colony PCR
pGEX-6P-1:2 _*Ab*PPO4_fwd	aGGAATTCCCATGTCTCTGCTCGCT ACTG	Correction of the reading frame for *Ab*PPO4
pGEX-6P-1:2 _rev	GGGGATCCCAGGGGCCCC	Correction of the reading frame for *Ab*PPO4
l*Ab*PPO4_fwd	TAAGGGTCGACTCGAGCG	Mutagenesis of *Ab*PPO4
l*Ab*PPO4_rev	AGTGAGACCGATGTTGACTAC	Mutagenesis of *Ab*PPO4: Δ(A566 – F611)
a*Ab*PPO4_rev	AGACTTCTTGATACCGTACCG	Mutagenesis of *Ab*PPO4: Δ(E384 – F611)
3C->EK_for	cgacaagGGACCAAACACAGAATTTG	Replacing the HRV 3C recognition sequence
3C->EK_rev	tcatcgtcATCCGATTTTGGAGGATG	Replacing the HRV 3C recognition sequence

Table S2: Data collection and refinement statistics for recombinant *Ab*PPO4

Parameter	Unit	Value
**X-Ray source**		ID23-1 (ESRF)
**Crystal Data**		
space group		C 1 2 1
unit cell: a, b, c	Å	287.3, 52.09, 152.66
unit cell: α, β, γ	°	90, 98.03, 90
molecules per asymmetric unit		4
maximal resolution	Å	3.25
Wilson B-factor	$Å^2$	97.41
**Data collection and processing**		
wavelength	Å	0.873
resolution range	Å	48.9 - 3.25 (3.366 - 3.25)
total reflections		150295 (15278)
unique reflections		35520 (3462)
Redundancy		4.2 (4.4)
R_{merge} [a]	%	0.2511 (1.108)
R_{meas} [b]	%	0.2879 (1.261)
$R_{p.i.m.}$ [c]	%	0.1383 (0.5916)
$CC_{1/2}$ [d]		0.994 (0.826)
Completeness	%	98.40 (98.49)
$\langle I/\sigma_I \rangle$		5.68 (1.91)
**Refinement statistics**		
used resolution range	Å	48.9 - 3.25
used reflections		35512 (3460)
reflections for calculation of R_{free}		1775 (173)
R_{work} [e]	%	0.2509 (0.3702)
R_{free} [f]	%	0.2752 (0.3827)
average B-factor	$Å^2$	144.53
r.m.s.d. of bond lengths	Å	0.004
r.m.s.d. of bond angles	°	0.70
**Ramachandran plot** [g]		
favored	%	94.29
allowed	%	5.43
outliers	%	0.28
**Rotamer outliers**	%	0.00
**PDB ID**		5M6B

Values in parantheses are for the highest-resolution shell.

a: $R_{merge} = \sum_{hkl}\sum_i |I_i(hkl) - \langle I(hkl)\rangle| \,/\, \sum_{hkl}\sum_i \langle I(hkl)\rangle$, where $I_i(hkl)$ is the ith observation of the intensity of reflex hkl and $\langle I(hkl)\rangle$ is the weighted average intensity for all symmetry-related observations of reflection hkl.

b: $R_{meas} = \sum_{hkl}\left(\frac{N_i}{N_i-1}\right)^{1/2} \sum_{hkl}\sum_i |I_i(hkl) - \langle I(hkl)\rangle| \,/\, \sum_{hkl}\sum_i \langle I(hkl)\rangle$, where N_i is the number of independent observations of reflection hkl.

c: $R_{p.i.m.} = \sum_{hkl}\left(\frac{1}{N_i-1}\right)^{1/2} \sum_{hkl}\sum_i |I_i(hkl) - \langle I(hkl)\rangle| \,/\, \sum_{hkl}\sum_i \langle I(hkl)\rangle$.

d: $CC_{1/2}$ is the Pearson's correlation coefficient between two halfs of the (randomly split) dataset.

e: $R_{work} = \sum_{hkl}|F(hkl)_{obs} - F(hkl)_{calc}| \,/\, \sum_{hkl} F_{obs}$, where $F(hkl)_{obs}$ and $F(hkl)_{calc}$ are the observed and the calculated structure factor amplitude for the reflection hkl, respectively.

f: R_{free} is R_{work} calculated for a randomly chosen 5 % of the reflections in the dataset which were not used for refinement.

g: calculated with Coot.[15]

Figures

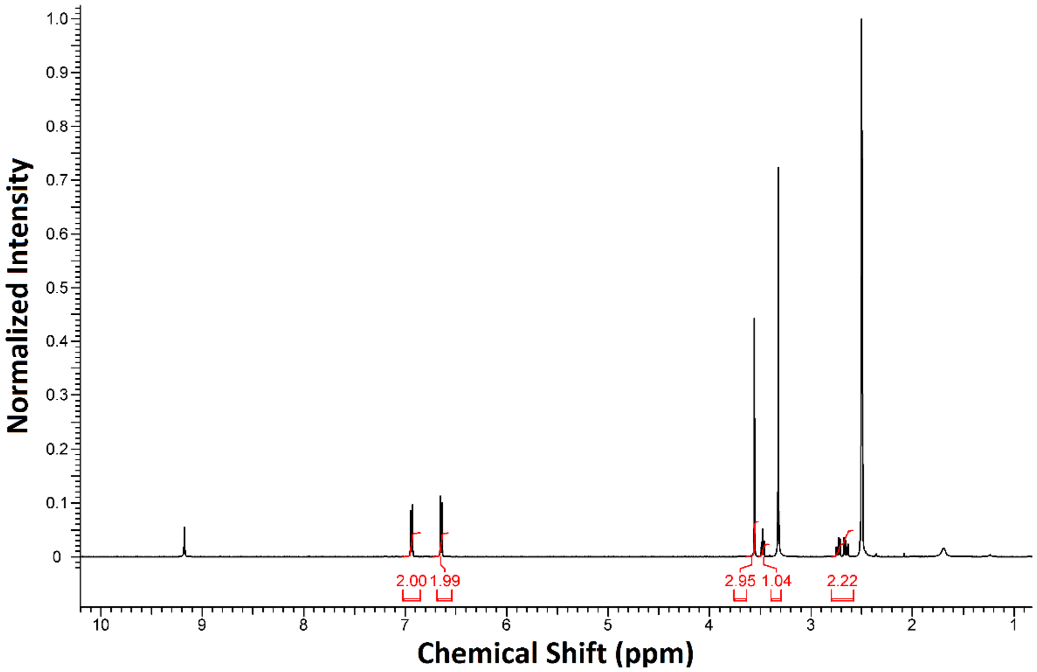

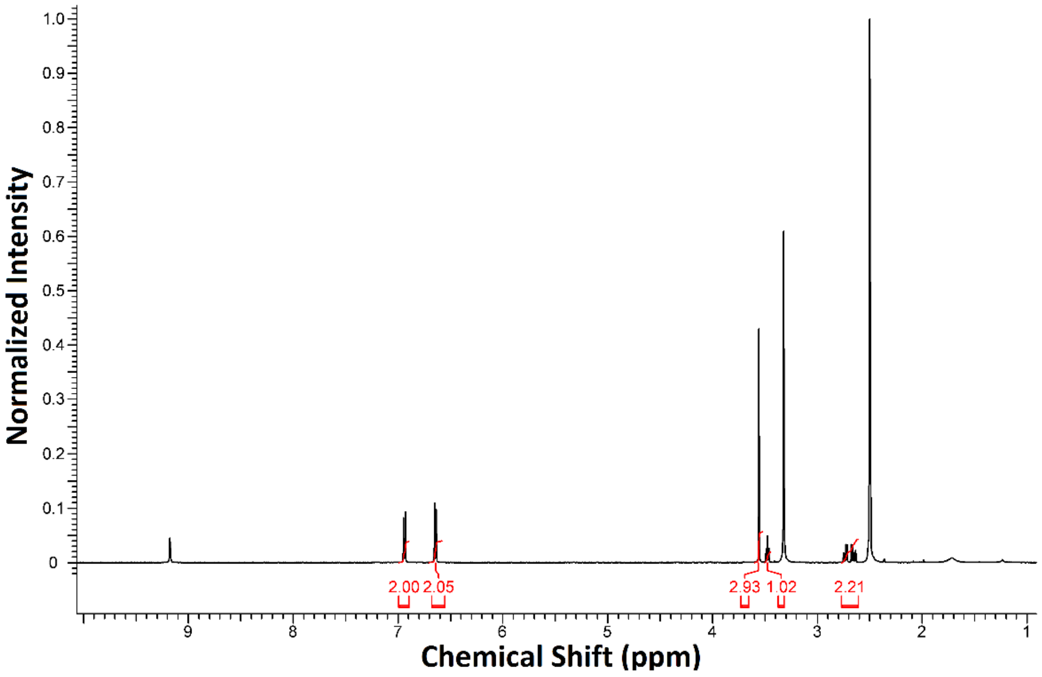

Figure S1: ^{1}H NMR (500 MHz, d_6-DMSO) of tyrosine methyl esters

Top: *L*-tyrosine methyl ester, bottom: *D*-tyrosine methyl ester

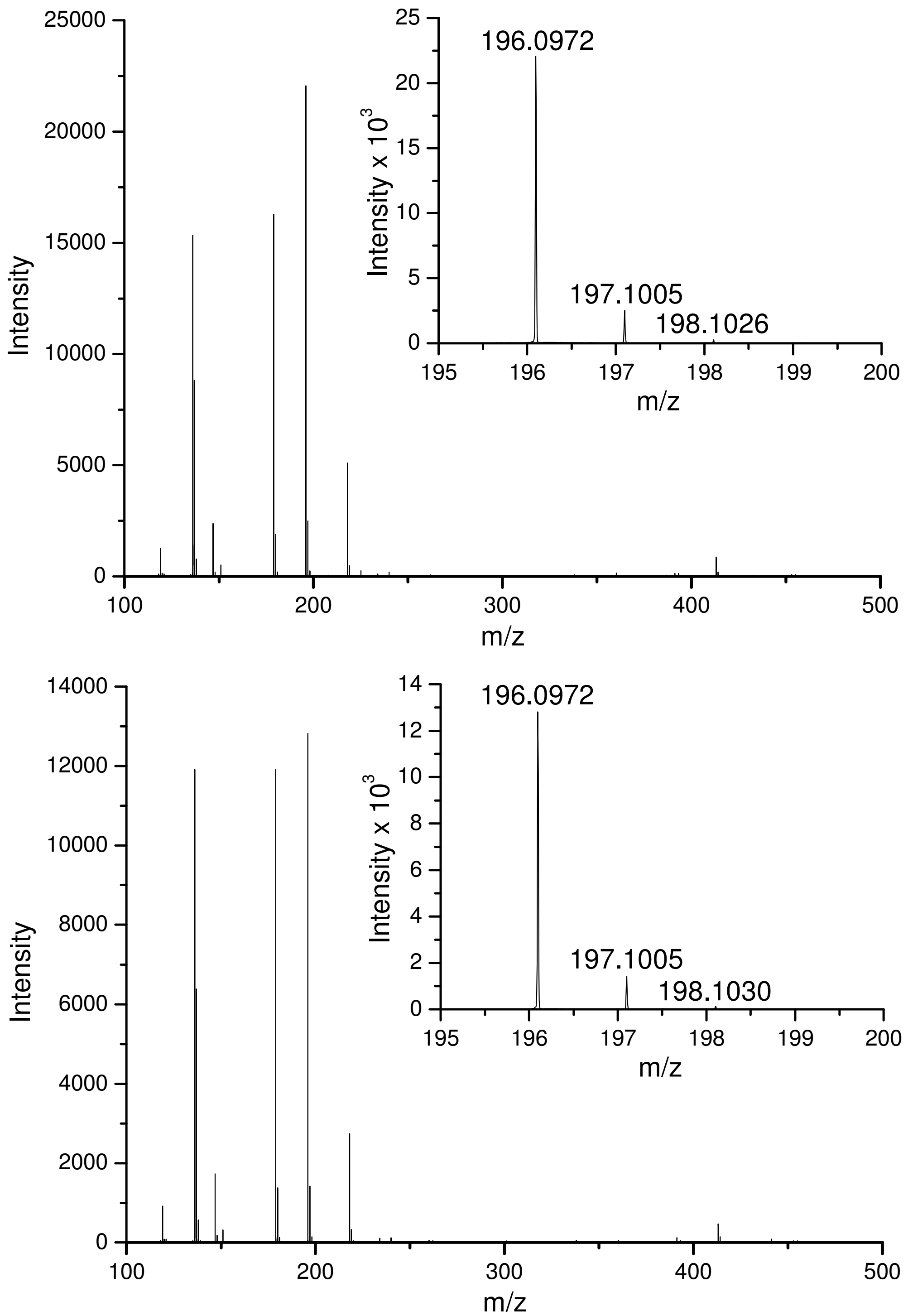

Figure S2: +ESI-MS spectrum of *D*-tyrosine methyl ester

Top: *L*-tyrosine methyl ester, bottom: *D*-tyrosine methyl ester

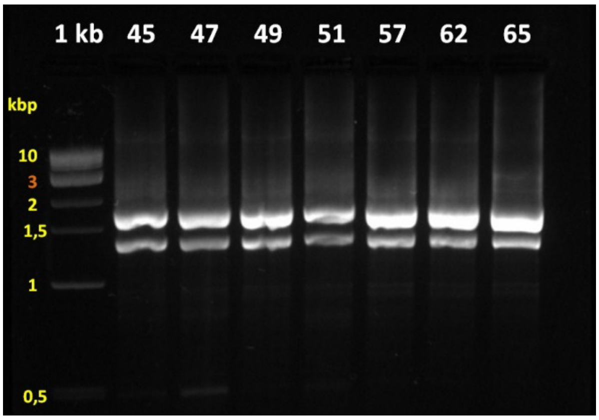

Figure S3: PCR with *Taq* DNA-polymerase on *A. bisporus* cDNA

The pair of primers used was *Ab*PPO4_fwd and *Ab*PPO4_rev. 1 kb marks the molecular size standard, numbers above the lanes indicate the primer annealing temperature (in °C) that was used in the PCR cycles for the respective sample. The 1.2 % agarose gel was run for 80 min at 6.8 V cm^{-1} and DNA was detected using the fluorescence of SYBR® Safe DNA stain (Fisher Scientific, Vienna, Austria) excited at 302 nm.

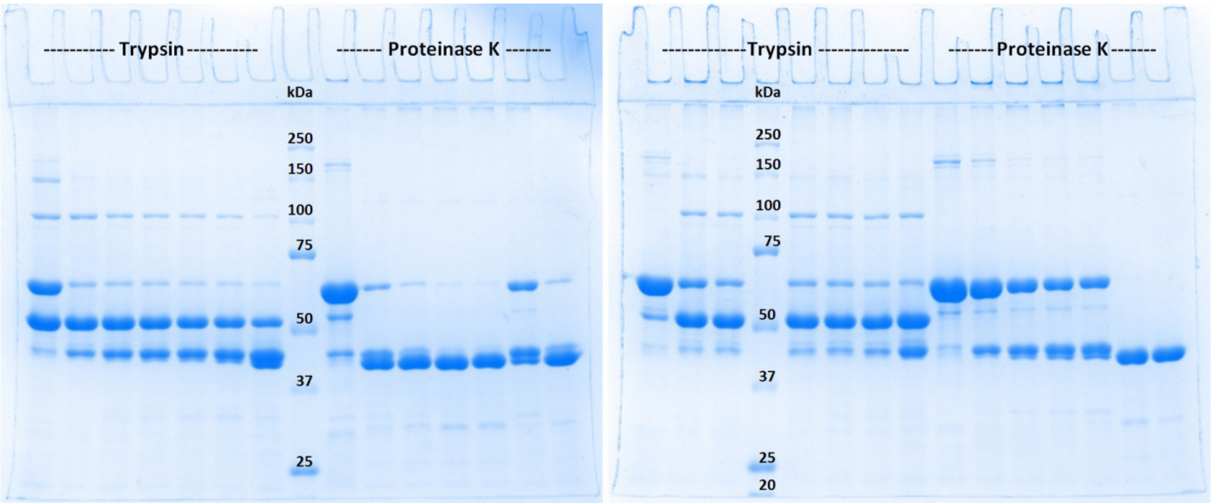

Figure S4: Proteolytic activation of latent *Ab*PPO4: SDS-PAGE

The gels show the activation of latent *Ab*PPO4 by the action of trypsin or proteinase K at different reaction times (from left to right: 0 min, 15 min, 30 min, 45 min, 1 h, 2 h and 18 h for each of the two proteases respectively) and with different ratios of protease to latent tyrosinase (left gel: molar ratio of 1 to 1, right gel: 1 to 10). The sample for 15 min reaction time with proteinase K at a ratio of 1 to 1 (left gel) was placed between the 2 h and 18 h samples. The lanes indicated with "kDa" contain the molecular weight marker; the size of the standard bands is given in kDa.

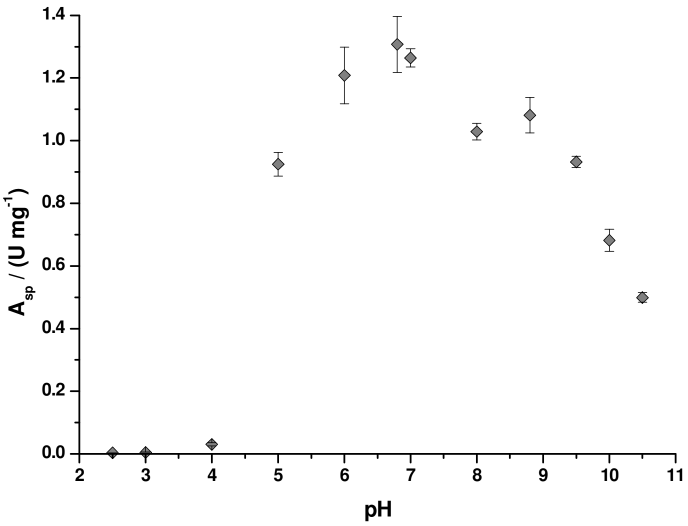

Figure S5: pH-dependence of the reaction rate of activated *Ab*PPO4 on 1 mM *L*-tyrosine in 50 mM sodium citrate

The reaction rates in buffers with pH > 7 were scaled by the relative reaction rate in 50 mM of the respective buffer substance (pH ≤ 7: Na-citrate, pH 8 - 8.8: Tris-HCl, pH 9.5 - 10.5: Glycin/NaOH) set to pH 7 and 50 mM sodium citrate buffer at pH 7 as the reference system. Reaction rates without enzyme at the respective pH were subtracted from each data point, but the autooxidation rates of tyrosine were not significantly different from zero even at pH 10.5. One data point represents the average of three measurements, the error bars show ± one standard deviation.

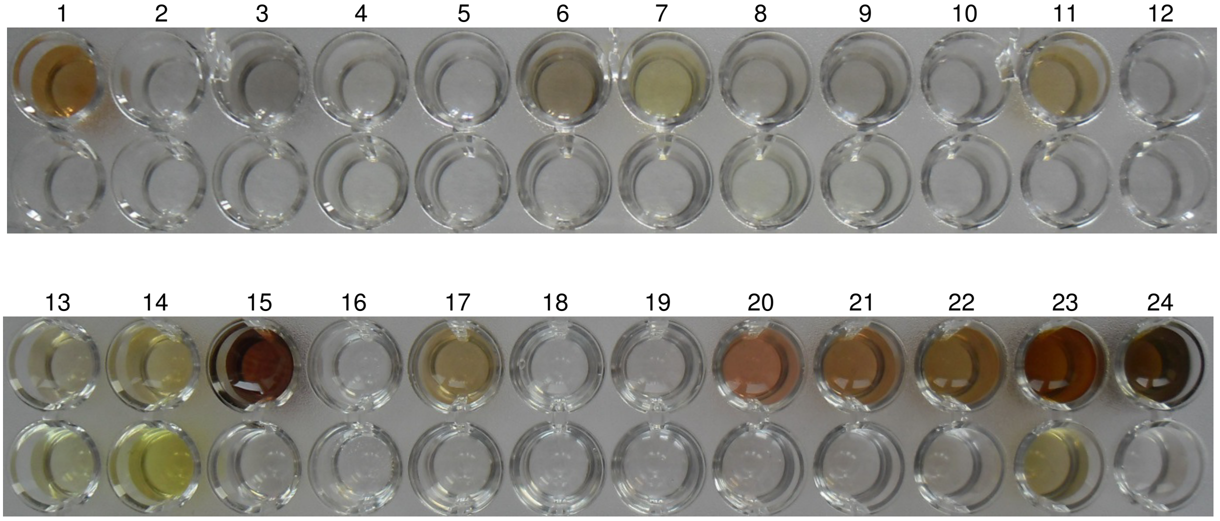

Figure S6: substrate acceptance of activated *Ab*PPO4

The reaction solutions in the top row contain activated *Ab*PPO4 while in the lower row the substrate solutions were incubated without addition of enzyme. Enzymatic reactions were carried out in a total volume of 200 µl containing 50 mM sodium citrate buffer pH 6.8, 4 µg activated *Ab*PPO4 (or the equivalent volume of water in the lower row) and the substrates at concentrations specified in the table below. Enzymatic activities are given in a qualitative manner: no discernable activity (-), change in color visible after 2 s (+++), after ≤ 30 s (++) and after 30 s - 30 min (+).

Well	Substrate	Concentration / mM	Activity
1	p-Tyrosol	5	++
2	2-Naphthol	0.5	-
3	Chlorogenic acid	0.5	+++
4	Fisetin	0.1	+*
5	Ferulic acid	0.5	-
6	p-Coumaric acid	0.5	++
7	4-*tert*-Butylcatechol#	1	+++
8	Quercetin	0.1	+*
9	Rutin	0.1	+
10	Kaempferol	0.1	-
11	Naringenin	0.1	++
12	DMSO°	28	-

Well	Substrate	Concentration / mM	Activity
13	Isoliquiritigenin	0.1	+++
14	Butein	0.1	+++
15	Octopamine	5	++
16	Thymol	5	-
17	4-Methylcatechol	1	+++
18	Vanillic acid	5	-
19	Resorcinol	5	+
20	Hydroquinone	5	+
21	Protocatechuic acid	1	++
22	3,4-Dihydroxy-phenylacetic acid	1	+++
23	Pyrogallol	5	+++
24	3-Methoxyphenol	5	+

*: The tyrosinase reaction on these flavanols proceeded in an unusual way: fast darkening followed by almost immediate loss of color and a slow reformation of a second colored product.

#: The reaction on the respective monophenol took longer than 30 min for the development of visible color. Addition of hydrogen peroxide (at a final concentration of 2.25 mM) increased the reaction rate on 4-*tert*-Butylphenol to the ++ level.

°: solvent blank.

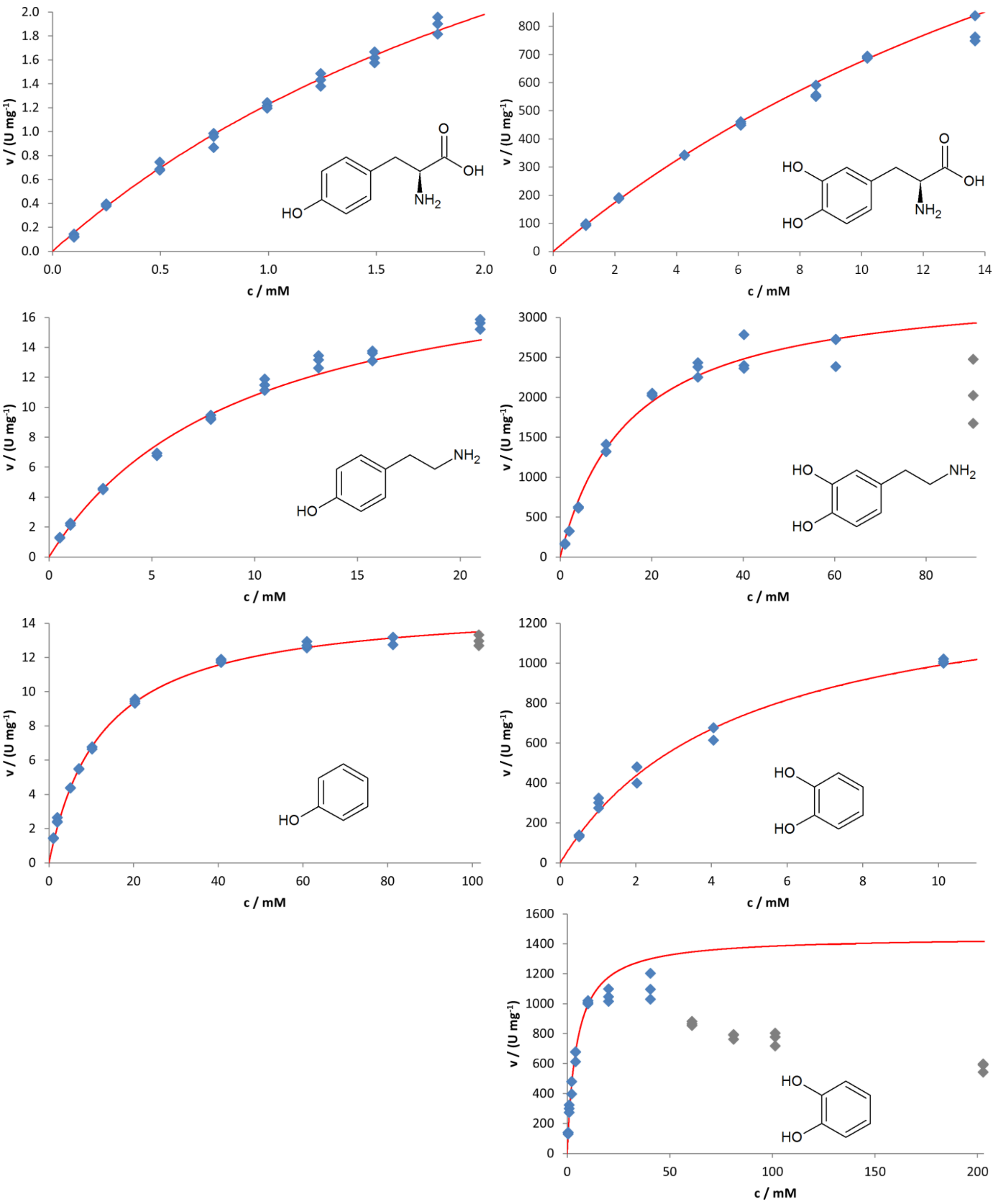

Figure S7: Michaelis-Menten diagrams for the substrates in Table 2

Top left: *L*-Tyrosine, top right: *L*-DOPA; middle left: tyramine, middle right: dopamine; bottom left: phenol, bottom right: catechol (2 diagrams showing the region of substrate concentration in which the Michaelis-Menten model is applicable and the total substrate concentration range tested). Blue and grey diamonds represent measured slopes, data point marked with grey diamonds indicate a significant contribution of substrate inhibition. Those data points were excluded from the data analysis yielding the least-squares optimized parameters of the Michaelis-Menten models. Reaction rates predicted using those models are shown as red curves.

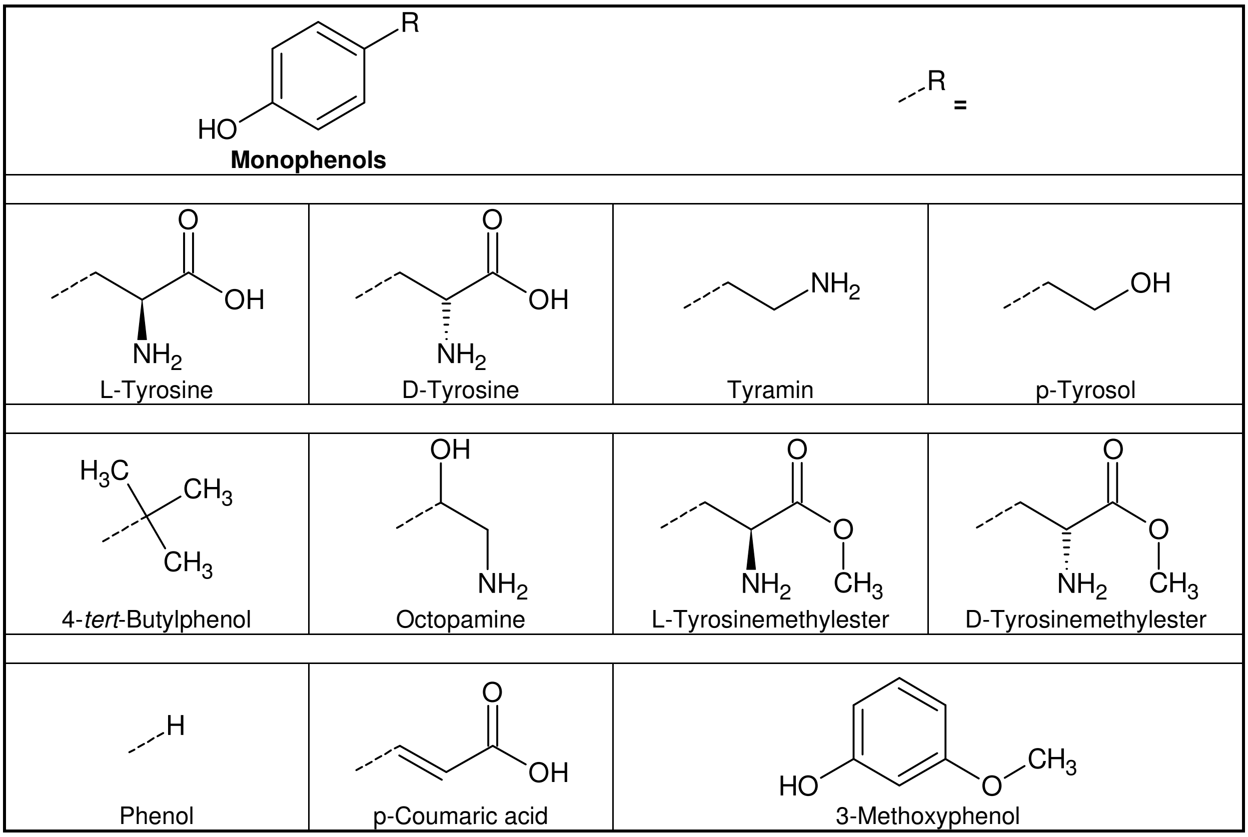
R
HO
Monophenols
R =
O
OH
NH2
L-Tyrosine
D-Tyrosine
Tyramin
p-Tyrosol
H3C
CH3
CH3
4-tert-Butylphenol
Octopamine
L-Tyrosinemethylester
D-Tyrosinemethylester
H
Phenol
p-Coumaric acid
3-Methoxyphenol

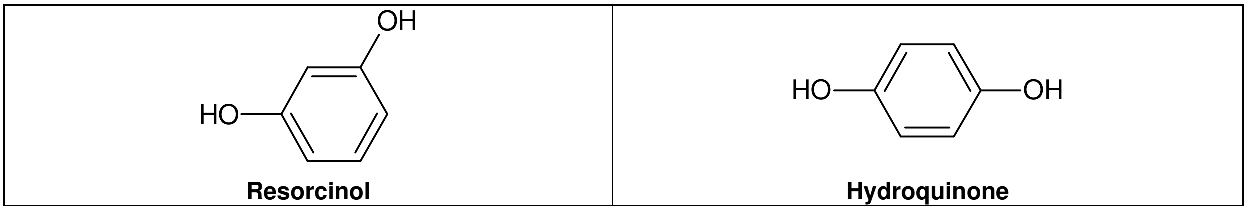
OH
HO
Resorcinol
Hydroquinone

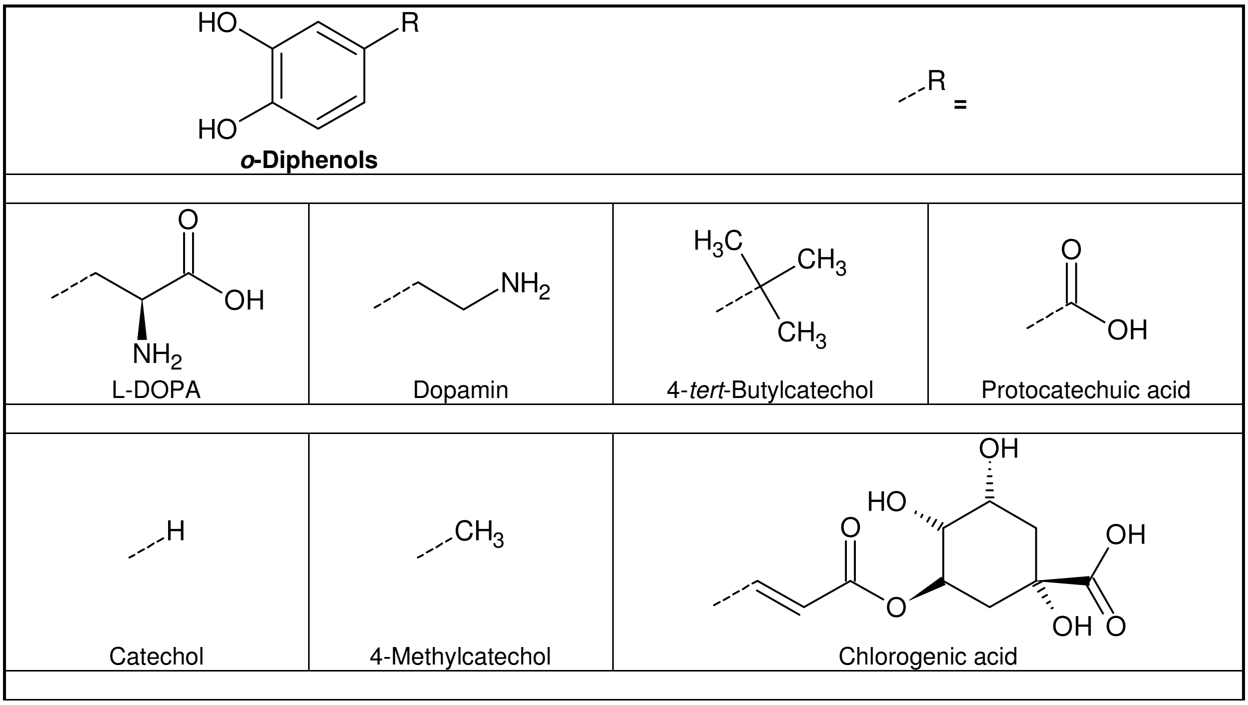
HO
R
o-Diphenols
R =
O
OH
NH2
L-DOPA
Dopamin
H3C
CH3
4-tert-Butylcatechol
Protocatechuic acid
H
Catechol
4-Methylcatechol
Chlorogenic acid

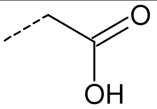<br>3,4-Dihydroxyphenylacetic acid	

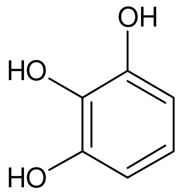<br>**Pyrogallol**	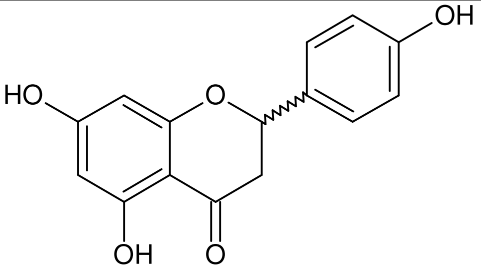<br>**Naringenin** (a flavanone)

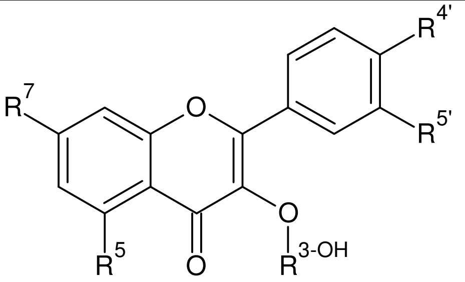<br>**Flavonols**	**Fisetin**	$R^{3\text{-OH}}$ = H, R^5 = H, R^7 = OH, $R^{4'}$ = OH, $R^{5'}$ = OH
	**Quercetin**	$R^{3\text{-OH}}$ = H, R^5 = OH, R^7 = OH, $R^{4'}$ = OH, $R^{5'}$ = OH
	**Rutin**	$R^{3\text{-OH}}$ = 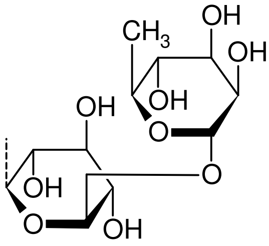 , R^5 = OH, R^7 = OH, $R^{4'}$ = OH, $R^{5'}$ = OH

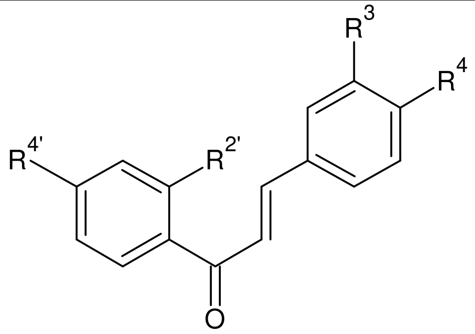<br>**Chalcons**	**Isoliquiritigenin**	R^3 = H, R^4 = OH, $R^{2'}$ = OH, $R^{4'}$ = OH
	**Butein**	R^3 = OH, R^4 = OH, $R^{2'}$ = OH,$R^{4'}$ = OH

Figure S8: Structures of substrates accepted by activated *Ab*PPO4

References

1. Hammond, J. B. W., Nichols, R. Carbohydrate Metabolism in *Agaricus bisporus* (Lange) Sing.: Changes in Soluble Carbohydrates during Growth of Mycelium and Sporophore. *J. Gen. Microbiol.* **93**, 309–320, (1976).

2. Weijn, A., Bastiaan-Net, S., Wichers, H. J., Mes, J. J. Melanin biosynthesis pathway in *Agaricus bisporus* mushrooms. *Fungal Genet. Biol.* **55**, 42–53, (2013).

3. Mülhardt, C. 2013. Der Experimentator Molekularbiologie / Genomics. In: Mülhardt, C, editor. Springer Spektrum. Experimentator 7, pp. 140–142.

4. Hanahan, D. Studies on transformation of Escherichia coli with plasmids. *J. Mol. Biol.* **166**, 557–580, (1983).

5. Mülhardt, C. 2013. Der Experimentator Molekularbiologie / Genomics. In: Mülhardt, C, editor. Springer Spektrum. Experimentator 7, pp. 154–155.

6. Hofmann, M. A., Brian, D. A. Sequencing PCR DNA amplified directly from a bacterial colony. *Biotechniques* **11**, 30–31, (1991).

7. Studier, F. W. Protein production by auto-induction in high-density shaking cultures. *Protein Expr. Purif.* **41**(1), 207–234, (2005).

8. Dirks-Hofmeister, M. E., Kolkenbrock, S., Moerschbacher, B. M. Parameters That Enhance the Bacterial Expression of Active Plant Polyphenol Oxidases. *PLoS ONE* **8**, e77291, (2013).

9. Sher, A., Veber, M., Marolt-Gomišček, M. Spectroscopic and polarographic investigations: Copper(II)-penicillin derivatives. *Int. J. Pharm.* **148**, 191–199, (1997).

10. French, C. S., Milner, H.W. 1955. [9] Disintegration of bacteria and small particles by high-pressure extrusion. In: Academic Press. Methods in Enzymology, Vol. 1, pp. 64–67.

11. McCoy, A. J., Grosse-Kunstleve, R. W., Adams, P. D., Winn, M. D., Storoni, L. C., Read, R. J. Phaser crystallographic software. *J. Appl. Crystallogr.* **40**, 658–674, (2007).

12. Mauracher, S. G., Molitor, C., Al-Oweini, R., Kortz, U., Rompel, A. Latent and active *ab*PPO4 mushroom tyrosinase cocrystallized with hexatungstotellurate(VI) in a single crystal. *Acta Crystallogr. Sect. D* **70**, 2301–2315, (2014).

13. Winn, M. D., Ballard, C. C., Cowtan, K. D, Dodson, E. J., Emsley, P., Evans, P. R., Keegan R. M., Krissinel, E. B., Leslie, A. G. W., McCoy, A., McNicholas, S. J., Murshudov, G. N., Pannu, N. S., Potterton, E. A., Powell, H. R., Read, R. J., Vagin, A., Wilson, K. S. Overview of the CCP4 suite and current developments. *Acta Crystallogr. Sect. D* **67**, 235–242, (2011).

14. Adams, P. D., Afonine, P. V., Bunkóczi, G., Chen, V. B., Davis, I. W., Echols, N., Headd, J. J., Hung, L.-W., Kapral, G. J., Grosse-Kunstleve, R. W., McCoy, A. J., Moriarty, N. W., Oeffner, R., Read, R. J., Richardson, D. C., Richardson, J. S., Terwilliger, T. C., Zwart, P. H. PHENIX: a comprehensive Python-based system for macromolecular structure solution. *Acta Crystallogr. Sect. D* **66**, 213–221, (2010).

15. Emsley, P., Cowtan, K. Coot: model-building tools for molecular graphics. *Acta Crystallogr. Sect. D* **60**, 2126–2132, (2004).

16. Terwilliger, T. C., Grosse-Kunstleve, R. W., Afonine, P. V., Moriarty, N. W., Zwart, P. H., Hung, L.-W., Read, R. J., Adams, P. D. Iterative model building, structure refinement and density modification with the PHENIX AutoBuild wizard. *Acta Crystallogr. Sect. D* **64**, 61–69, (2008).
